# A distinct 2,2′-diphenic acid-mediated pyrene degradation pathway in a novel PAH degrader, *Glutamicibacter soli* ENR6

**DOI:** 10.1128/aem.02064-25

**Published:** 2026-03-03

**Authors:** Pengrui Zhang, Haiyang Zhang, Xinyu Yang, Yuanli Wu, Shanshan Dai, Yanqin Ding, Shanshan Sun

**Affiliations:** 1 College of Life Sciences and Shandong Engineering Research Center of Plant-Microbial Restoration for Saline-alkali Land, Shandong Agricultural University 34734, Tai’an, China; Universidad de los Andes, Bogotá, Colombia

**Keywords:** *Glutamicibacter soli*, pyrene, degradation pathway, 2,2′-diphenic acid, functional genes

## Abstract

Bacteria play an important role in the biodegradation of polycyclic aromatic hydrocarbons (PAHs), especially the more recalcitrant high-molecular-weight compounds like pyrene. In this study, we isolated and characterized a novel pyrene-degrading bacterium, *Glutamicibacter soli* ENR6, from petroleum-contaminated soil. Strain ENR6 was able to utilize various PAHs as sole carbon sources and degrade 88.8% of 50 mg L^−1^ pyrene and 76.8% of 100 mg L^−1^ pyrene within 15 days. During pyrene degradation, eight metabolites were detected, with 2,2′-diphenic acid emerging as the predominant intermediate. By day 7, 40.6% of the degraded pyrene had accumulated in the form of 2,2′-diphenic acid, which was subsequently metabolized via the phthalate pathway. To our knowledge, this is the first report identifying 2,2′-diphenic acid as an intermediate in pyrene degradation. Integrated genomic and transcriptomic analyses revealed four gene clusters (clusters I–IV) involved in pyrene degradation in strain ENR6. Reverse transcription-quantitative PCR (RT-qPCR) analysis demonstrated that these clusters were specifically induced by pyrene and its intermediates, with genes *chr_3400-chr_3404* in cluster I strongly associated with 2,2′-diphenic acid degradation. Based on these findings, we propose a distinct 2,2′-diphenic acid-centered pathway for pyrene degradation in strain ENR6. This study not only broadens the bacterial repertoire available for PAH bioremediation but also provides new insights into the metabolic diversity of PAH-degrading microorganisms.

**IMPORTANCE:**

Polycyclic aromatic hydrocarbons (PAHs) are widespread, highly toxic, and persistent environmental pollutants. Microbial biodegradation offers a sustainable approach for PAH remediation, yet the metabolic mechanisms remain incompletely understood. In this study, we isolated and characterized a novel pyrene-degrading bacterium, *Glutamicibacter soli* ENR6, thereby expanding the bacterial repertoire available for PAH bioremediation. The identification of 2,2′-diphenic acid as a key intermediate reveals a previously unrecognized pathway of pyrene degradation, in which the subsequent catabolism of 2,2′-diphenic acid is mediated by a newly identified ring-hydroxylating dioxygenase. This pathway provides an alternative metabolic entry point into the broadly conserved phthalate pathway when the classical 1-hydroxy-2-naphthoic acid-centered pathway is inhibited. The distinct 2,2′-diphenic acid-mediated pyrene degradation pathway elucidated in strain ENR6 highlights metabolic diversity among PAH-degrading microorganisms and provides a mechanistic foundation for applying this strain in the bioremediation of PAH-contaminated sites.

## INTRODUCTION

Polycyclic aromatic hydrocarbons (PAHs) are a class of widespread and recalcitrant organic pollutants. They are primarily derived from the incomplete combustion of organic matter, including fossil fuels, biomass, and various industrial processes (1, 2). Due to their hydrophobicity, structural stability, and carcinogenic/mutagenic properties, PAHs tend to accumulate in both terrestrial and aquatic environments, posing significant threats to ecosystems and human health (3, 4). In particular, high-molecular-weight (HMW) PAHs, which consist of four or more aromatic rings, are more resistant to degradation and exhibit greater toxicity, making their remediation especially challenging (5, 6).

Microbial biodegradation is widely regarded as an efficient and eco-friendly strategy for the detoxification and mineralization of PAHs. Over the past decades, various bacterial genera, such as *Mycobacterium*, *Pseudomonas*, and *Shinella,* have been shown to possess the ability to degrade PAHs (7–9). Among them, *Arthrobacter* species have frequently been reported to degrade low-molecular-weight (LMW) PAHs (e.g., naphthalene, phenanthrene) (10–12). *Glutamicibacter*, a genus reclassified from *Arthrobacter* in 2016 (13), is commonly found in soil and rhizosphere environments and is primarily known for its halotolerance and plant growth-promoting traits (14, 15). However, despite its close phylogenetic relationship with *Arthrobacter*, no *Glutamicibacter* strain has been reported to degrade PAHs to date. Furthermore, a recent comparative genomic study has revealed that *Glutamicibacter* and *Arthrobacter* differ markedly in their xenobiotic degradation potential (16). Although *Arthrobacter* species are well documented for their ability to degrade phenanthrene, the catabolic pathways and genetic determinants underlying PAH degradation remain largely unknown in *Glutamicibacter*.

As a model compound of HMW-PAHs, pyrene degradation pathways have been intensively investigated in several bacteria, such as *Mycobacterium* and *Cycloclasticus* strains (8, 17, 18). In these organisms, the primary mineralization pathway typically begins with a dioxygenase-catalyzed attack at the 4,5-carbon positions, leading to the formation of a key intermediate, 1-hydroxy-2-naphthoic acid (1H2N). This compound is further metabolized through two major “lower pathways”: the salicylate and phthalate pathways, and serves as a central metabolic node in the degradation of both pyrene and phenanthrene (18–21). While 2,2′-diphenic acid (DIPA) has frequently been reported as an *ortho*-cleavage product of 9,10-dihydroxyphenanthrene during phenanthrene degradation, it is generally not considered as a major intermediate in this process (19, 22). For example, a study on *Mycobacterium* sp. WY10 revealed that only approximately 2% of the phenanthrene (100 mg L^−1^) was converted into DIPA, indicating its limited role in the catabolic pathway (19). Moreover, to our knowledge, the subsequent breakdown of DIPA has not been well characterized in the literature.

Here, we reported the isolation and characterization of a novel PAH-degrading bacterium, *Glutamicibacter soli* ENR6, from petroleum-contaminated soil. By integrating metabolite profiling, genome sequencing, transcriptomic analysis, and reverse transcription-quantitative PCR (RT-qPCR) validation, we elucidated the metabolic pathways and key gene clusters involved in pyrene degradation by strain ENR6. Notably, our findings reveal a distinct degradation pathway in which DIPA serves as the predominant intermediate. This study expands the bacterial resource for PAH biodegradation and provides new insights into the metabolic diversity of PAH-degrading organisms.

## MATERIALS AND METHODS

### Chemicals and culture media

The following reagents were obtained from Sigma-Aldrich (Shanghai, China): fluorene (98%), phenanthrene (98%), pyrene (98%), and BSTFA-TMCS [*N,O*-bis (trimethylsilyl) trifluoroacetamide-trimethylchlorosilane, 99:1, vol/vol]. Standards including 1H2N (98%), DIPA (98%), salicylic acid (≥99%), phthalic acid (≥99%), and protocatechuic acid (≥97%) were sourced from Aladdin (Shanghai, China). All organic solvents used in this work were of high-performance liquid chromatography grade. Mineral salts medium (MSM) was used for bacterial isolation and degradation assays (19, 20), while Luria-Bertani (LB) broth served for strain culture. Inorganic reagents used were of analytical grade or higher.

### Isolation and characterization of pyrene-degrading strains

Soil samples were collected from a petroleum-contaminated site near oil wells in Shandong, China. The enrichment and isolation procedures followed methods described in our previous studies (20, 23). After three enrichment cycles, the culture was diluted and spread onto MSM agar plates containing 250 mg L^−1^ pyrene. One isolate exhibiting a large and clear degradation zone was obtained and designated as strain ENR6.

For taxonomic identification of the isolated strain, colony morphology and Gram staining characteristics were recorded. Cell morphology was examined using a field-emission scanning electron microscope (FE-SEM, GeminiSEM 300, ZEISS, Germany) after platinum sputter coating. Genomic DNA of strain ENR6 was extracted using a bacterial genomic DNA isolation kit (Sangon Biotech, Shanghai, China). The 16S rRNA gene was amplified with universal primers 27F (5′-AGAGTTTGATCCTGGCTCA-3′) and 1492R (5′-CGGTTACCTTGTTACGACTT-3′), and the PCR product was subsequently sequenced. A phylogenetic tree was constructed based on the 16S rRNA gene sequence using the neighbor-joining method in MEGA 11 software.

### Identification of intermediate metabolites

The isolated strain ENR6 was pre-cultured in LB broth until the late exponential growth phase, then harvested by centrifugation and resuspended in sterile MSM to an OD_600_ of 1.0 (approximately 3.37 × 10⁸ CFU mL^−1^). Ten milliliters of the cell suspension was inoculated into 90 mL MSM supplemented with either pyrene (100 mg L^−1^), 1H2N (50 mg L^−1^), or DIPA (50 mg L^−1^) as the sole carbon source. Cultures were incubated at 30°C with shaking at 180 rpm for 5 days (for pyrene) or 4 days (for 1H2N and DIPA). Cultures sampled at day 0 served as controls. All experiments were performed in triplicate.

Metabolite extraction was performed according to our previously published methods (19, 20). A Shimadzu GCMS-QP 2010 S system equipped with an Rtx-5MS column (60 m × 0.25 mm i.d., 0.25 μm film thickness) was used for mass spectral analysis. Helium served as the carrier gas at a constant flow rate of 2.0 mL min^−1^. Samples (1 μL) were injected in splitless mode. Other conditions, including the oven temperature program, injector and detector settings, and mass spectrometric acquisition parameters, were identical to those described in our previous study (19).

### Quantification of pyrene degradation and intermediate metabolites

Quantitative analysis of fluorene (50 mg L^−1^), phenanthrene (50 mg L^−1^), or pyrene (50, 60, 100, and 300 mg L^−1^) degradation by strain ENR6 was conducted in 50-mL Erlenmeyer flasks containing 10 mL of MSM. The inoculum volume and incubation conditions were consistent with those described in “Identification of intermediate metabolites,” above. Sterile MSM without bacterial inoculation served as the abiotic control. At predefined time points, triplicate samples were collected, and residual pyrene, along with its metabolites, was extracted following the protocol of Sun et al. (24).

Analyses were performed using a Waters ACQUITY UPLC H-Class system equipped with a PDA detector and a reversed-phase column C_18_ (ACQUITY UPLC BEH C_18_, 1.7 µm, 2.1 × 100 mm). The elution program was slightly modified based on the methods described by Sun et al. (19, 24). The mobile phases consisted of 0.1% formic acid in acetonitrile (solvent A) and 0.1% formic acid in water (solvent B). The flow rate was set at 0.3 mL min^−1^. A gradient elution was applied as follows: solvent A was initially set at 8%, increased to 14% at 3 min, to 39% at 10 min, to 60% at 15 min, to 75% at 17 min, and finally returned to 8% at 20 min. The column temperature was maintained at 30°C and the injection volume was 10 μL. Under these conditions, protocatechuic acid, phthalic acid, salicylic acid, DIPA, 1H2N, phenanthrene, and pyrene were successfully separated and detected in a single run (19). Under this method, 1H2N could be reliably detected at concentrations as low as 20 μg L^−1^, which represents its limit of detection.

The degradation of pyrene and the formation/consumption of DIPA were described using a branched first-order kinetic model. The system was represented by the following coupled differential equations:


$$\frac{dS}{dt}=-k_{tot}S,$$

$$\frac{dP}{dt}=f\;k_{tot}S-k_{2}P,$$

$$k_{a}=f\;k_{tot},$$

where *S* (mg L^−1^) and *P* (mg L^−1^) are the concentrations of pyrene and DIPA, respectively. The parameters are total pyrene degradation rate constant, *k*_tot_ (d^−1^), branching fraction representing the proportion of pyrene converted to DIPA, *f*, DIPA degradation rate constant, *k*_2_ (d^−1^), and the effective rate constant for DIPA formation, *k*_a_ (d^−1^). Thus, higher *k*_tot_ and *k*_2_ values correspond to faster pyrene degradation and DIPA consumption, whereas an elevated *f* indicates the dominance of the DIPA-centered pathway. The coupled equations were globally fitted to the experimental time-course data of pyrene and DPA concentrations. Model fitting was performed in R using the *deSolve* and *minpack.lm* packages. Parameter uncertainty was evaluated by parametric bootstrap (500 resamplings), providing 5%–95% percentile intervals. The bootstrap distribution was also used to assess the robustness of the estimated branching fraction *f*.

### Genome sequencing and analysis

Genome sequencing of strain ENR6 was conducted using Illumina MiSeq combined with PacBio Sequel platforms (Shanghai Personalbio Technology Co., Ltd.). Quality filtering of raw reads (AdapterRemoval and SOAPEC), genome assembly and polishing (HGAP, CANU, and Pilon), protein-coding gene prediction (GeneMarkS and RAST), and functional annotation (NR, KEGG, eggNOG, and Swiss-Prot) were performed as previously described (25–27). The average nucleotide identity (ANI) between strain ENR6 and related strains was calculated using JSpeciesWS (http://jspecies.ribohost.com/jspeciesws). Homologous sequence alignment between strain ENR6 and *Arthrobacter* sp. YC-RL1 was visualized using MAUVE.

### Transcriptome sequencing and analysis

Culture samples were collected after 4 days of growth with pyrene (100 mg L^−1^) and after 10 h of growth with glucose (10 mmol L^−1^), at which point the cell density of both treatments reached OD_600_ = 0.2 ± 0.03. Total RNA was extracted using a previously established TRIzol-based protocol (28). The concentration, purity, and integrity of RNA extracted from triplicate cultures of each treatment were assessed (28). Strand-specific libraries were then constructed using the ALFA-SEQ RNA Library Prep Kit II (Findrop Biosafety Technology, Guangzhou, China), and sequenced on the Illumina NovaSeq 6000 platform. After removing ribosomal RNA reads, transcript sequences were aligned to the genome of strain ENR6 using Bowtie2 (v2.4.5). Gene-level read counts were obtained with RSEM (v1.3.3), and expression levels were normalized as transcripts per million (TPM). Differentially expressed genes (DEGs) between treatments were identified using DESeq2 (v1.34.0), with thresholds of false discovery rate (FDR) ≤0.05 and |log₂ (fold change)| ≥1. Gene Ontology (GO) and Kyoto Encyclopedia of Genes and Genomes (KEGG) enrichment analyses of DEGs were performed using the ClusterProfiler package (v4.2.2), with significantly enriched terms defined as those with FDR ≤0.05.

### Reverse transcription-quantitative PCR analysis

Culture samples grown with pyrene (100 mg L^−1^) and glucose (10 mmol L^−1^, as the control) were collected as described in “Transcriptome sequencing and analysis,” above. Additional samples were harvested after 3 days of growth with individual metabolites, including DIPA, 1H2N, salicylic acid, phthalic acid, and protocatechuic acid (Fig. S1). Each metabolite was supplied at 50 mg L^−1^. First-strand cDNA was synthesized from 1 μg of total RNA using the Evo M-MLV RT Kit with gDNA removal (Accurate Biotechnology, Hunan, China). RT-qPCR was performed on a Rotor-Gene Q real-time PCR system (Qiagen, Germany) using the SYBR Green Pro Taq HS qPCR Kit (Accurate Biotechnology, Hunan, China). All oligonucleotides used for RT-qPCR analysis were custom-synthesized (Table S1). The amplification program was as follows: initial denaturation at 94°C for 30 s; followed by 40 cycles of 94°C for 5 s, 55°C for 20 s, and 72°C for 20 s; and a final extension at 72°C for 5 min. Relative gene expression levels were calculated using the 2^−ΔΔCt^ method (29), with 16S rRNA gene as the internal reference gene. The expression level of the internal reference gene was normalized to 1.0. RT-qPCR on samples from three biological replicates, each analyzed in three technical replicates.

## RESULTS

### *Glutamicibacter soli* ENR6 was isolated from soil and could degrade various PAHs

**Fig 1:**
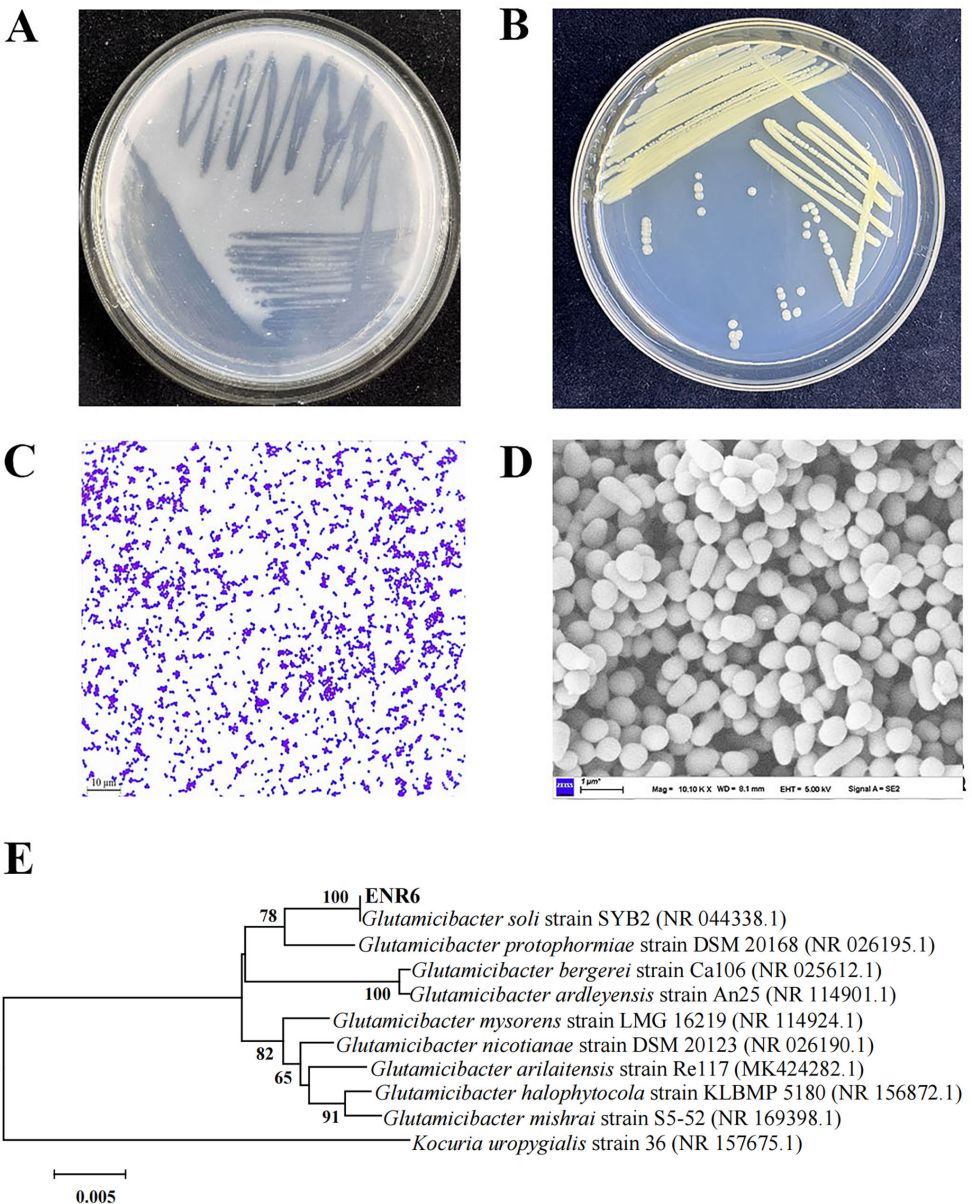
Identification and characterization of the pyrene-degrading bacterium strain ENR6. Colony morphology of strain ENR6 on (**A**) MSM plate supplemented with 250 mg L^−1^ pyrene and on (**B**) LB plate. Cell morphology observed by (**C**) Gram staining under light microscopy (1,000×), and by (**D**) FE-SEM (10,000×). (**E**) Phylogenetic tree was constructed based on the 16S rRNA gene sequence by neighbor-joining methods. The numbers on the branch nodes indicate the percentages of bootstrap support for the clades based on 1,000 bootstrap resamplings.

A bacterial colony, designated strain ENR6, was isolated from petroleum-contaminated soil for its ability to grow on an MSM plate containing pyrene (250 mg L^−1^) as the sole carbon source. After 7 days of incubation, a clear degradation zone surrounding the colony was observed, indicating its efficient pyrene-degrading ability (Fig. 1A). On the LB agar plate, colonies appeared light yellow and opaque, with regular margins and a smooth, moist surface (Fig. 1B). Morphological characterization revealed that strain ENR6 was a Gram-positive, short rod-shaped bacterium (Fig. 1C and D). The nearly full-length 16S rRNA gene sequence of strain ENR6, consisting of 1,399 nucleotides, was obtained and exhibited 99.9% similarity to that of *Glutamicibacter soli* strain SYB2 (NR 044338.1). Phylogenetic analysis based on the 16S rRNA gene placed strain ENR6 in the same subclade as strain SYB2 (Fig. 1E). Therefore, strain ENR6 was tentatively identified as *Glutamicibacter soli* ENR6.

**Fig 2:**
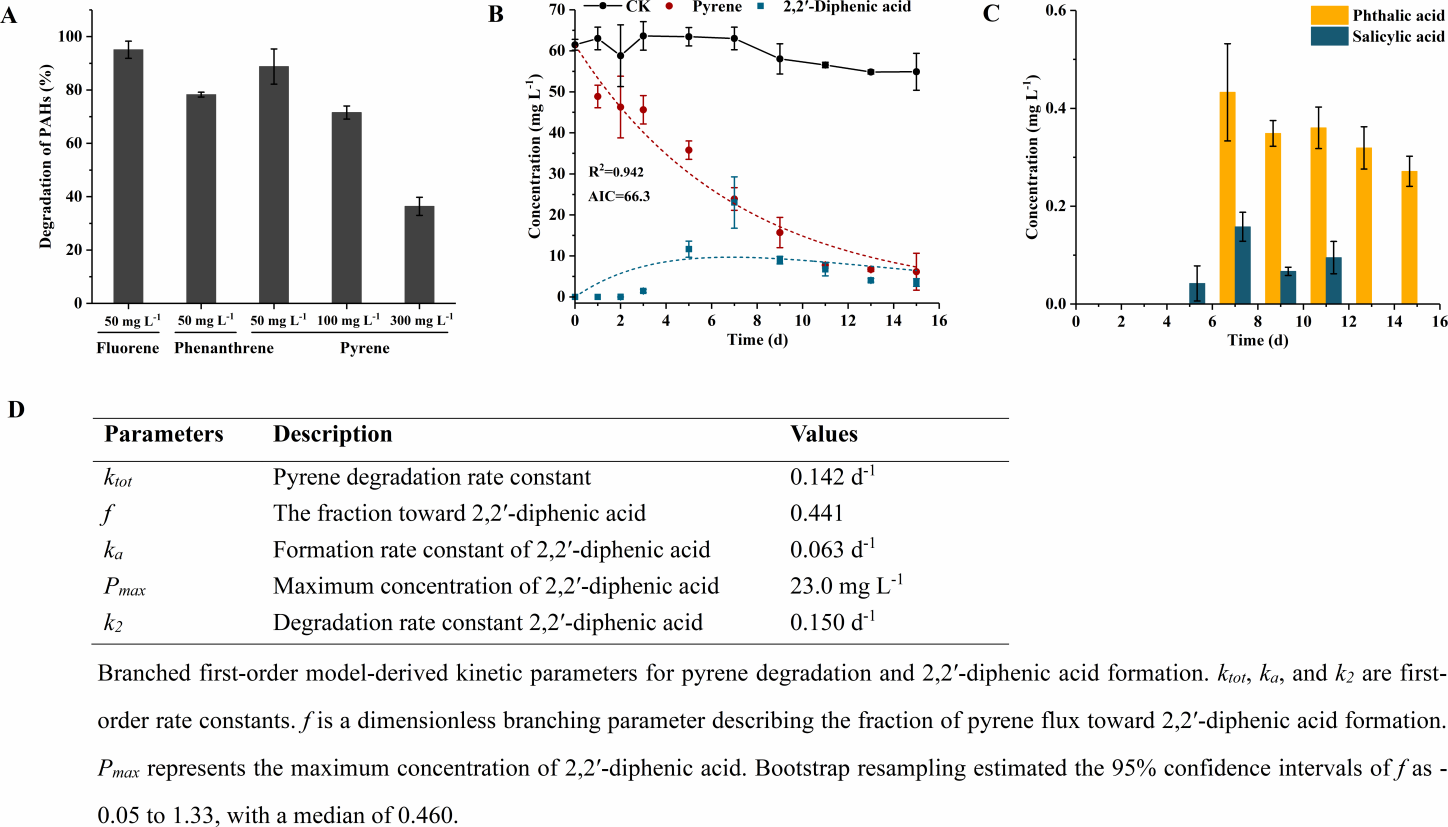
Degradation ability of *Glutamicibacter soli* ENR6 toward PAHs and metabolite dynamics during pyrene degradation. (**A**) Degradation of fluorene (3 days), phenanthrene (7 days), and pyrene (15 days) by strain ENR6. (**B**) Pyrene (60 mg L^−1^) degradation kinetics and DIPA production kinetics over 15 days. (**C**) Production kinetics of phthalic acid and salicylic acid during pyrene degradation. (**D**) The parameters of a branched first-order model for pyrene degradation and DIPA production.

Strain ENR6 was capable of utilizing various PAHs as the sole carbon source. It degraded 95.1% of fluorene within 3 days and 78.3% of 50 mg L^−1^ phenanthrene within 7 days. Moreover, ENR6 exhibited substantial activity toward the HMW-PAH pyrene, degrading 88.8%, 76.8%, and 36.4% of 50, 100, and 300 mg L^−1^ pyrene, respectively, after 15 days of incubation (Fig. 2A).

### *Glutamicibacter soli* ENR6 generated a novel product, 2,2′-diphenic acid, during pyrene degradation

**Fig 3:**
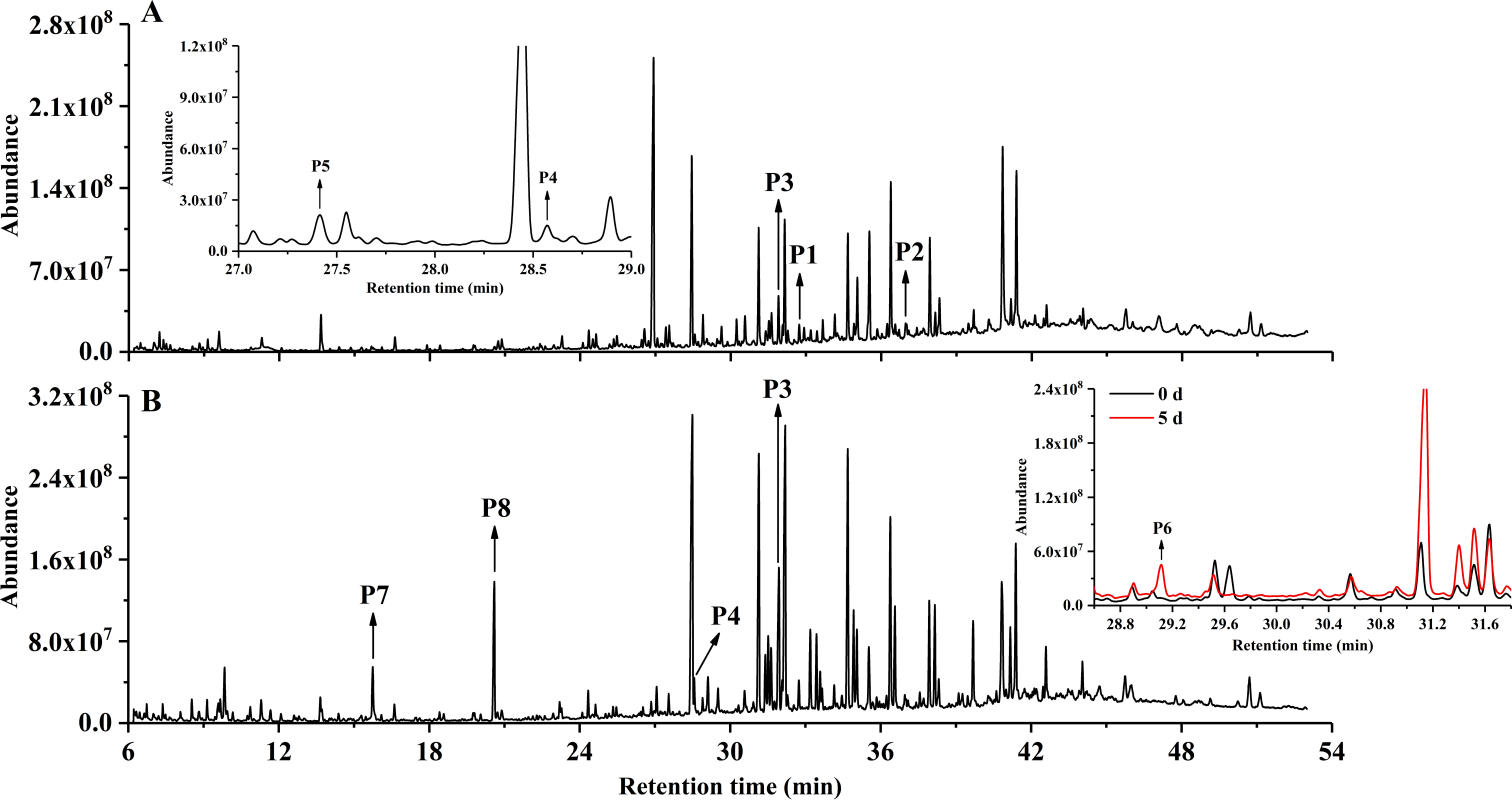
GC-MS chromatograms of (**A**) neutral and (**B**) acidic metabolites treated with BSTFA-TMCS after 5 d of pyrene (100 mg L^−1^) degradation by *Glutamicibacter soli* ENR6.

In addition to several peaks corresponding to linear-chain acids and derivatization reagent adducts, eight metabolites (P1–P8) were identified during the degradation process by strain ENR6 (Table 1; Fig. 3). Metabolites P1–P4, with retention times of 32.93, 37.43, 31.91, and 28.57 min, were identified as the TMS derivatives of pyrene *cis*-4,5-dihydrodiol, 4,5-dihydroxypyrene, 4-phenanthrenecarboxylic acid, and 4-phenanthrol, respectively, consistent with our previous reports (19, 23). Metabolite P5, exhibiting an *m/z* of 196, showed a mass spectrum identical to that of 7,8-benzocoumarin as previously reported (30). Notably, in the acidic fraction, a metabolite (P6) was detected at a retention time of 29.12 min (Fig. 3). By comparing its retention time and mass spectrum with those of the authentic standard, P6 was identified as DIPA (Fig. S2). To our knowledge, this is the first report of DIPA being detected as an intermediate in pyrene degradation. Two metabolites, P7 and P8, were confirmed as salicylic acid and phthalic acid, respectively, also by comparison with authentic standards (Table 1).

**TABLE 1:** GC retention times (RT) and mass spectral data of intermediate metabolites during the degradation of pyrene, 2,2'-diphenic acid, or 1-hydroxy-2-naphthoic acid

Peak^*a*^	Fraction^*b*^	RT	*m/z* of fragment ions (% relative intensity)	Identified metabolite
Pyrene as the sole carbon source				
P1	N	32.93	380 (M^+^, 37), 365 (M^+^-CH_3_, 29), 290 (M^+^-OTMS-H^+^, 77), 218 (M^+^-OTMS-TMS, 4), 202 (M^+^−2OTMS, 22), 147 (2TMS+H^+^, 99), 73 (TMS, 100)	*cis*-4,5-pyrene dihydrodiol (diTMS)
P2	N	37.43	378 (M^+^, 96), 290 (M^+^-TMS-CH_3_, 39), 260 (M^+^-TMS-3CH_3_, 11), 73 (TMS, 100)	4,5-Dihydroxypyrene (diTMS)
P3	A/N	31.91	294 (M^+^, 70), 279 (M^+^-CH_3_, 58), 205 (M^+^-OTMS, 100), 177 (M^+^-COOTMS, 49), 151 (11), 73 (TMS, 24)	4-Phenanthrenecarboxylic acid (TMS)
P4	A/N	28.57	266 (M^+^, 100), 251 (M^+^-CH_3_, 67), 235 (M^+^−2CH_3_-H^+^, 74), 165 (5), 73 (TMS, 35)	4-Phenanthrol (TMS)
P5	N	27.42	196 (M^+^, 100), 168 (M^+^-CO, 99), 139 (M^+^-CO-CHO, 62)	7,8-Benzocoumarin
P6	A	29.12	371 (M^+^-CH_3_, 5), 269 (M^+^-TMS-2CH_3_-CH_2_, 100), 178 (10), 147 (2TMS+H^+^, 50), 73 (TMS, 89)	2,2'-Diphenic acid (diTMS)
P7	A	15.74	267 (M^+^-CH_3_, 92), 209 (M^+^-TMS, 12), 193 (M^+^-OTMS, 7), 149 (M^+^-OTMS-2CH_3_-CH_2_, 9), 135 (M^+^-2TMS-H^+^, 6), 91 (M^+^−2TMS-COOH, 7), 73 (TMS, 100)	Salicylic acid (diTMS)
P8	A	20.59	310 (M^+^, 11), 295 (M^+^-CH_3_, 95), 221 (M^+^-OTMS, 57), 163 (M^+^-2TMS-H^+^, 14), 147 (2TMS+H^+^, 100), 73 (TMS, 100)	Phthalic acid (diTMS)
2,2′-Diphenic acid as the sole carbon source				
M1	N	26.29	196 (M^+^, 100), 168 (M^+^-CO, 43), 139 (M^+^-CO-CHO, 41)	3,4-Benzocoumarin
P8	A/N	20.59	310 (M^+^, 11), 295 (M^+^-CH_3_, 95), 221 (M^+^-OTMS, 57), 163 (M^+^-2TMS-H^+^, 14), 147 (2TMS+H^+^, 100), 73 (TMS, 100)	Phthalic acid (diTMS)
1-Hydroxy-2-naphthoic acid as the sole carbon source				
M2	N	22.82	231 (M^+^-TMS-H^+^, 100), 216 (M^+^-OTMS, 71), 200 (M^+^-OTMS-CH_3_-H^+^, 38), 185 (M^+^-OTMS-2CH_3_-H^+^, 23), 73 (TMS, 75)	1,2-Naphthalenediol (diTMS)
M3	A	13.60	146 (M^+^, 84), 118 (M^+^-CO, 100), 90 (M^+^-CO-CHO+H^+^, 37), 89 (M^+^-CO-CHO, 36)	Coumarin
P7	A	15.74	267 (M^+^-CH_3_, 92), 209 (M^+^-TMS, 12), 193 (M^+^-OTMS, 7), 149 (M^+^-OTMS-2CH_3_-CH_2_, 9), 135 (M^+^-2TMS-H^+^, 6), 91 (M^+^-2TMS-COOH, 7), 73 (TMS, 100)	Salicylic acid (diTMS)
P8	A	20.59	310 (M^+^, 11), 295 (M^+^-CH_3_, 95), 221 (M^+^-OTMS, 57), 163 (M^+^−2TMS-H^+^, 14), 147 (2TMS+H^+^, 100), 73 (TMS, 100)	Phthalic acid (diTMS)

^*a*^ Metabolite identification corresponding with structures in Fig. 4. Metabolites P6, P7, and P8 were confirmed by comparing their retention times and mass spectra with those of authentic standards.

^*b*^ Fraction, acidic (A) or neutral (N), from which intermediate metabolites were detected.

**Fig 4:**
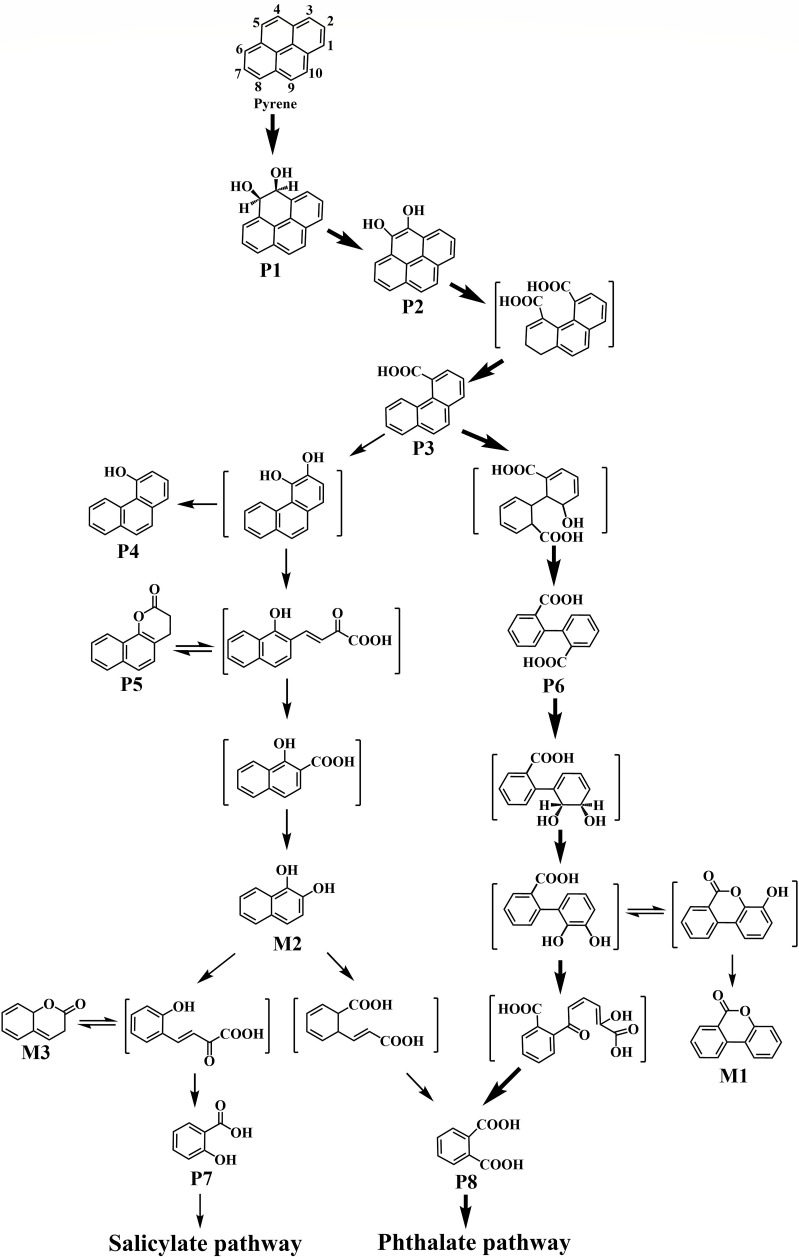
Proposed pyrene degradation pathways by *Glutamicibacter soli* ENR6. Metabolites in brackets were proposed, but not detected. Thick and thin arrows represent major and minor pathways, respectively.

Previously, DIPA was considered a dead-end metabolite during phenanthrene degradation (19, 31). However, our investigation revealed that strain ENR6 was capable of further degrading DIPA to form phthalic acid (P8) (Table 1; Fig. S3). An additional benzocoumarin-like compound (M1) was also detected in the DIPA degradation culture. It exhibited a retention time distinct from that of metabolite P5 (7,8-benzocoumarin) and was determined to be 3,4-benzocoumarin (22). Meanwhile, strain ENR6 was able to utilize 1H2N as the sole carbon source (Fig. S1), producing 1,2-naphthalenediol (M2), coumarin (M3), salicylic acid (P7), and phthalic acid (P8) (Table 1; Fig. S4). Altogether, these results suggest that a distinct pyrene degradation pathway was identified in strain ENR6, where degradation proceeds via DIPA to phthalate pathway, and via 1H2N to both salicylate and phthalate pathways (Fig. 4).

### *Glutamicibacter soli* ENR6 primarily degraded pyrene to phthalate pathway via 2,2′-diphenic acid as the central intermediate

When strain ENR6 was incubated with pyrene (60 mg L^−1^) as the sole carbon source, kinetics of accumulated metabolites during pyrene degradation were monitored. Among the detected intermediates, DIPA was the most abundant. Its concentration increased gradually, reaching 23.0 mg L^−1^ on day 7, at which point 47.3 mg L^−1^ of pyrene had been degraded, with 40.6% of the depleted pyrene accumulated as DIPA (Fig. 2B). After day 7, the DIPA concentration began to decline, coinciding with the appearance of its downstream product, phthalic acid. By day 15, DIPA had decreased to 3.53 mg L^−1^. In contrast, salicylic acid was present only at trace levels, reaching maximum concentration on day 7 and falling below the detection limit by day 13 (Fig. 2C). Consistent with the GC-MS analyses, 1H2N was not detected during pyrene degradation. These results suggested that 2,2′-diphenic acid was the major intermediate and a key node in pyrene degradation pathway by strain ENR6.

Further analysis showed that the pyrene degradation data were well described by the global fit of a branched first-order model, with an R^2^ value of 0.942. The total degradation rate constant of pyrene (*k*_tot_) was 0.142 d^−1^, corresponding to a half-life of 4.88 days for pyrene. The effective rate constant for DIPA formation (*k_a_*) was 0.063 d^−1^ (Fig. 2D). Notably, the DIPA degradation rate constant (*k_2_* = 0.150 d^−1^) was substantially higher than *k_a_*, indicating that DIPA consumption proceeded faster than its formation under the tested condition.

### Genomic and transcriptomic analyses reveal the aromatic hydrocarbon catabolic potential of strain ENR6

**Fig 5:**
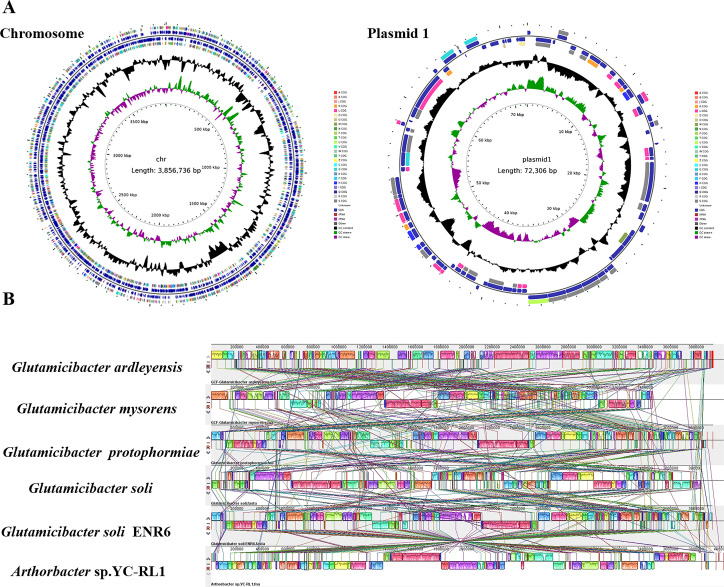
Genome sequencing and analysis of *Glutamicibacter soli* ENR6. (**A**) The circular maps of chromosome and plasmid DNA. (**B**) Genome comparison between strain ENR6 and closely related *Glutamicibacter* species and *Arthrobacter* sp. YC-RL1, visualized using Mauve. Homologous gene regions are shown as identically colored blocks, connected by lines.

The genome of strain ENR6 consists of a single circular chromosome of 3,856,736 bp and a small plasmid of 72,306 bp. A total of 3,657 genes were predicted, including 3,080 protein-coding genes, 66 tRNA genes, 19 rRNA genes, and 29 non-coding RNA genes (Fig. 5A; Table S2). Whole-genome ANI analysis revealed a 98.5% identity between strain ENR6 and *Glutamicibacter soli*, providing strong genomic evidence for its classification as *Glutamicibacter soli* ENR6 (Table S3). Genome comparison using MAUVE revealed a high degree of homology between the genome of strain ENR6 and that of *Glutamicibacter* species. Additionally, several gene fragments in strain ENR6 showed high sequence conservation with those of *Arthrobacter* sp. YC-RL1 (Fig. 5B), suggesting that the two strains may possess a common genetic framework supporting functions, such as the catabolism of aromatic hydrocarbons.

Sixty-six genes in the genome of strain ENR6 were predicted to be involved in the catabolism of aromatic hydrocarbons (Table S4). For example, a complete set of genes responsible for protocatechuate degradation was identified, enabling strain ENR6 to convert protocatechuate into tricarboxylic acid cycle intermediates via the *β*-ketoadipate pathway. This result was consistent with the observed ability of strain ENR6 to utilize protocatechuate as the sole carbon source (Fig. S1). To further identify the genes involved in pyrene degradation, a comparative transcriptomic analysis was conducted on strain ENR6 cells grown with pyrene versus glucose as the sole carbon source.

The gene expression profile of strain ENR6 grown on pyrene was distinct from that observed under glucose-grown conditions (Table S5; Fig. S5), indicating a carbon source-dependent transcriptional response and the induction of specific metabolic pathways associated with pyrene degradation. In total, 1,313 genes were differentially expressed, with 696 significantly upregulated (log₂FC > 1, FDR < 0.05) and 617 significantly downregulated (log₂FC < −1, FDR < 0.05) in cells grown with pyrene (Fig. S5A). Notably, 205 of the 1,313 differentially expressed genes were annotated as having unknown functions. Among the remaining genes, the major predicted functional categories based on eggNOG annotation included amino acid transport and metabolism [E], energy production and conversion [C], inorganic ion transport and metabolism [P], transcription [K], carbohydrate transport and metabolism [G], and lipid transport and metabolism [I] (Fig. S5B).

### Four gene clusters are predicted to be involved in pyrene biodegradation

**Fig 6:**
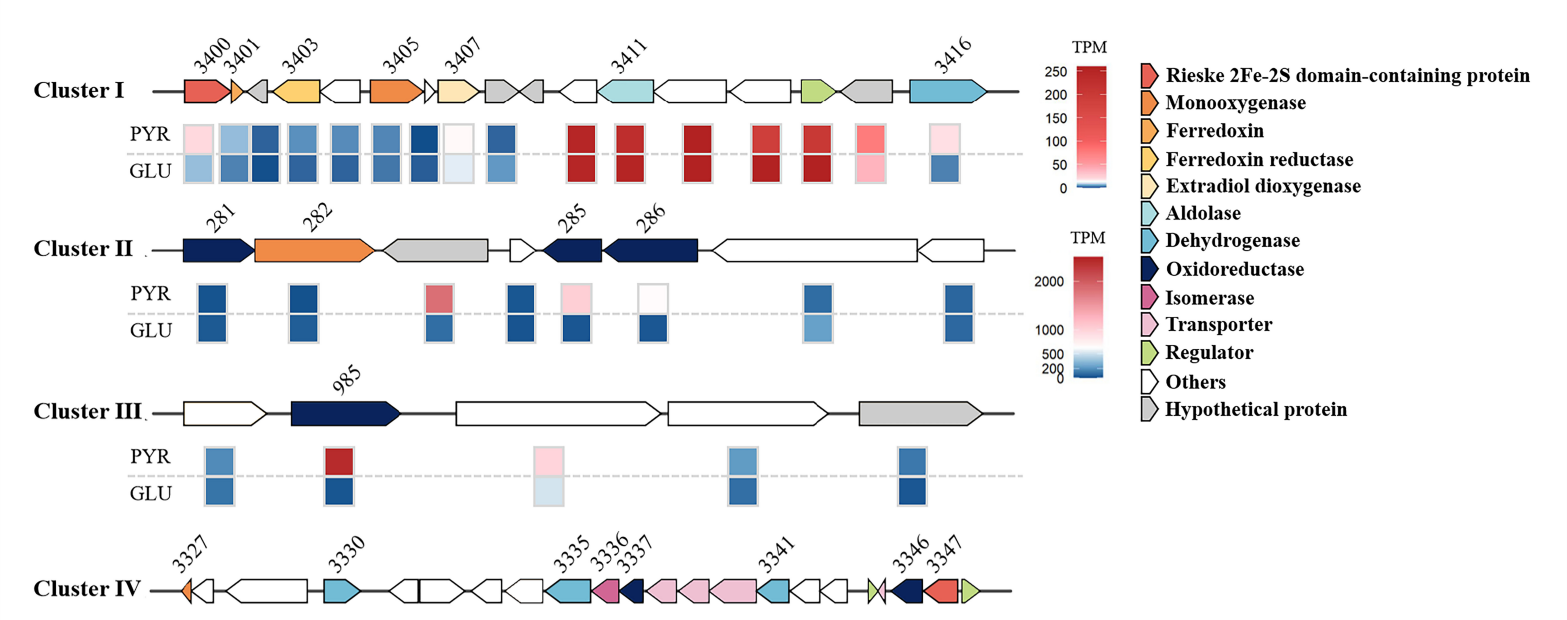
Putative pyrene degradation gene clusters in *Glutamicibacter soli* ENR6 and their expression during growth on pyrene (PYR) or glucose (GLU), based on TPM values. Data represent the averages from triplicate cultures. In the heatmap of cluster I, TPM values exceeding 260 are shown as dark red. Numbers above the arrows correspond to ORF locus tags from the ENR6 genome.

Integrated genomic and transcriptomic analyses identified four gene clusters (designated I–IV) as potential candidates involved in pyrene degradation (Fig. 6). Within cluster I, genes *chr_3400*, *chr_3403*, and *chr_3416* were significantly upregulated (over twofold, FDR < 0.05) in cells grown with pyrene compared to glucose. The predicted product of *chr_3400* is annotated as an oxygenase component containing a conserved [2Fe-2S] cluster motif. The predicted products of *chr_3401*, *chr_3403*, *chr_3411*, and *chr_3416* correspond to a ferredoxin, a ferredoxin-NADP^+^ reductase, an aldolase, and a dehydrogenase, respectively. In addition, *chr_3407* shares 100% sequence identity with *bphC*, a gene encoding 2,3-dihydroxybiphenyl 1,2-dioxygenase in *Arthrobacter* sp. YC-RL1 (10). Given that genes involved in aromatic hydrocarbon metabolism are often clustered together and their functions can frequently be inferred from genomic context (32, 33), genes *chr_3400-chr_3403* are proposed to encode an oxygenase and its associated electron transport components of a ring-hydroxylating dioxygenase (RHO).

This putative RHO may catalyze the ring hydroxylation of PAHs and other aromatic hydrocarbons, with Chr_3400 representing the α-subunit of the enzyme complex. To further examine this hypothesis, a phylogenetic tree was constructed using Chr_3400 along with 38 well-characterized RHO α-subunit sequences (Fig. S6A). The result showed that Chr_3400 clustered with BphA1 from *Pseudomonas pseudoalcaligenes* KF707, which has been identified as the oxygenase component of a three-component RHO system responsible for the hydroxylation of biphenyl/polychlorinated biphenyls (34). However, Chr_3400 shared less than 20% amino acid identity with BphA1 (Fig. S6B), suggesting that it may represent the α-subunit of a previously uncharacterized RHO.

In cluster II, pyrene exposure led to more than 20-fold upregulation of genes *chr_283*, *chr_285,* and *chr_286*. The predicted products of *chr_285* and *chr_286* were NADPH-dependent reductase and flavin-dependent oxidoreductase, respectively. Gene *chr_985,* located in cluster III, was remarkably upregulated and is predicted to encode NADPH-dependent oxidoreductase. In cluster IV, gene *chr_3347* is annotated as the α-subunit of an RHO. Analysis of the flanking nucleotide sequences revealed that genes *chr_3327*, *chr_3330*, *chr_3335*, *chr_3336, chr_3337*, *chr_3341*, and *chr_3346* are predicted to encode a monooxygenase, dehydrogenase, aldehyde dehydrogenase, isomerase, oxidoreductase, alcohol dehydrogenase, and an oxidoreductase, respectively (Fig. 6). However, transcriptomic analysis showed no detectable expression of these genes, which may be attributed to their extremely low transcriptional levels.

### Intermediate metabolites specifically induce different gene clusters as revealed by RT-qPCR analysis

**Fig 7:**
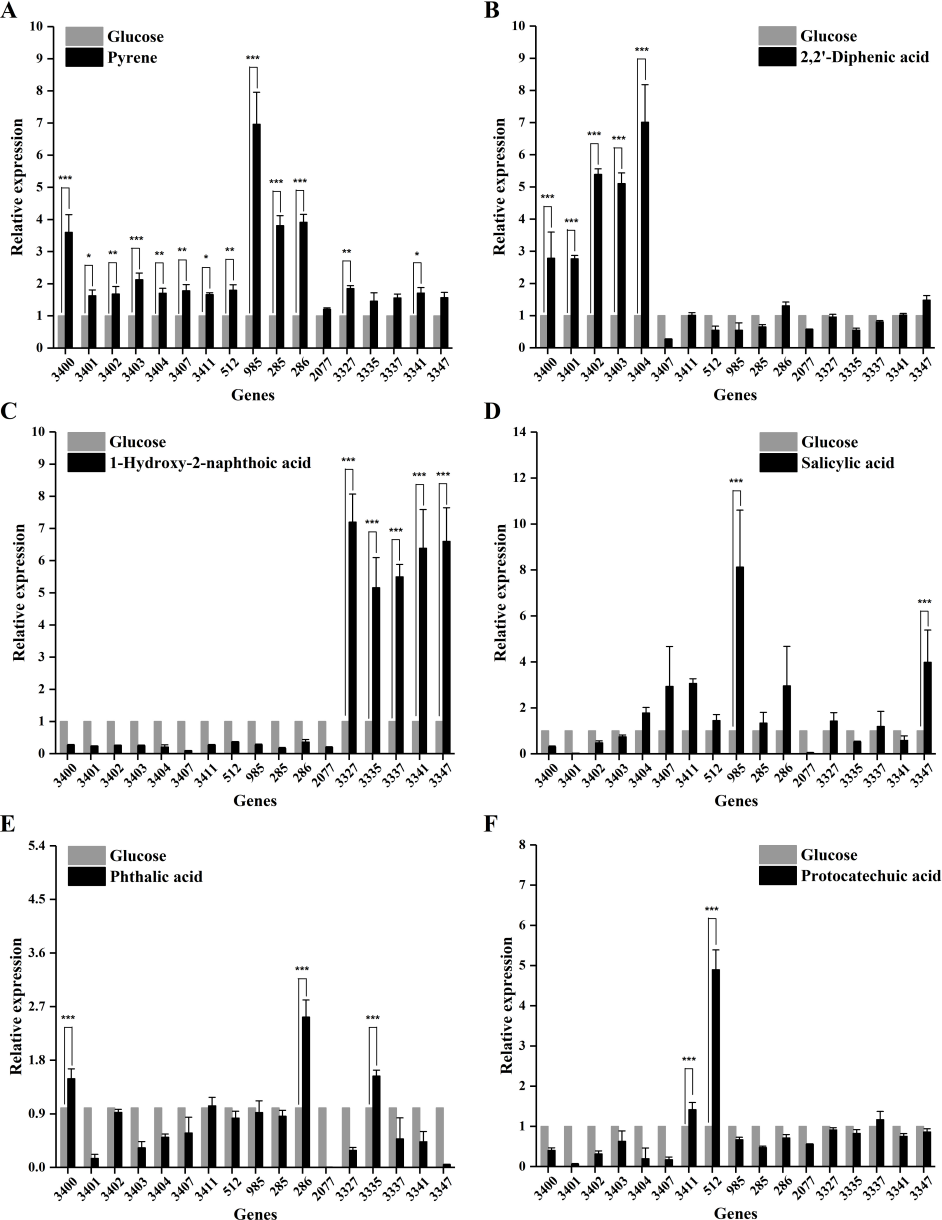
Transcriptional levels of putative pyrene-degrading genes in *Glutamicibacter soli* ENR6 by RT-qPCR analysis. Strain ENR6 was cultured in MSM supplemented with (**A**) 100 mg L^−1^ pyrene for 4 days, and (**B**) 50 mg L^−1^ 2,2′-diphenic acid, (**C**) 1-hydroxy-2-naphthoic acid, (**D**) salicylic acid, (**E**) phthalic acid, and (**F**) protocatechuic acid for 3 days. Cultures grown with 10 mmol L^−1^ glucose for 10 h served as the control.

To investigate the specific functions of the four gene clusters, the transcriptional levels of 17 associated genes were analyzed in strain ENR6 cells grown with pyrene or its intermediate metabolites as the sole carbon source. During growth with pyrene, the expression levels of genes *chr_3400* and *chr_3403* from cluster I, *chr_285* and *chr_286* from cluster II, and *chr_985* from cluster III were upregulated by more than twofold (*P* < 0.001), consistent with the transcriptomic analysis results. In addition, genes *chr_3401*, *chr_3402*, *chr_3407,* and *chr_3411* (cluster I), *chr_3327* and *chr_3341* (cluster IV), as well as *chr_512* predicted to encode an oxidoreductase, were also significantly expressed. In contrast, gene *chr_2077* that encodes a reductase iron-sulfur subunit was not significantly upregulated in both transcriptomic and RT-qPCR analysis during growth on pyrene (Fig. 7A).

Among the intermediate compounds, DIPA also strongly induced the expression of genes *chr_3400-chr_3404* in cluster I (Fig. 7B), highlighting their critical roles in the DIPA degradation pathway. In contrast, genes in cluster IV were markedly upregulated in response to 1H2N (Fig. 7C), suggesting their involvement in the transformation of 1H2N in strain ENR6. Moreover, the transcriptional levels of genes *chr_985* and *chr_3347* were upregulated by approximately ninefold and fourfold, respectively, in the presence of salicylic acid (Fig. 7D). Genes *chr_286* and *chr_3335* were upregulated in response to phthalic acid, while *chr_3411* and *chr_512* showed increased expression with protocatechuic acid (Fig. 7E and F). These findings suggest that the corresponding genes may be involved in the degradation of salicylic acid, phthalic acid, and protocatechuic acid, respectively.

## DISCUSSION

*Glutamicibacter* species were primarily recognized for its halotolerance and plant growth-promoting abilities (15). In this study, we provided the first evidence that a member of the *Glutamicibacter* genus is capable of degrading PAHs, thereby expanding the functional repertoire previously ascribed to this group. Although *Glutamicibacter* was reclassified from *Arthrobacter* in 2016 (13) and shared a close phylogenetic relationship with its parent genus, no *Glutamicibacter* strain had previously been reported to degrade PAHs. By contrast, certain *Arthrobacter* strains have been isolated and characterized for their ability to degrade LMW-PAHs (10–12). For example, *Arthrobacter* sp. YC-RL1, isolated from petroleum-contaminated soil, degraded over 90% of biphenyl (50 mg L^−1^) and naphthalene (50 mg L^−1^), and 87.9% of phenanthrene within 5 days (10). Similarly, *Arthrobacter* sp. SZ achieved 89% degradation of 50 mg L^−1^ phenanthrene within 10 days (12). In this work, strain ENR6 demonstrated the ability to degrade fluorene, phenanthrene, and pyrene, with pyrene being efficiently degraded at concentrations ranging from 50 to 300 mg L^−1^ (Fig. 2).

Nonetheless, recent comparative genomic analyses have revealed distinct differences in xenobiotic degradation potential between *Glutamicibacter* and *Arthrobacter* (16). The genome-based study involving 47 strains from both genera demonstrated that while *Arthrobacter* species are well documented for phenanthrene degradation, the genetic determinants and catabolic pathways responsible for PAH degradation remain underexplored in *Glutamicibacter*.

The identification of pyrene cis-4,5-dihydrodiol (P1), 4,5-dihydroxypyrene (P2), 4-phenanthrenecarboxylic acid (P3), and 4-phenanthrol (P4) indicated that strain ENR6 oxidized pyrene through the well-established “upper pathway.” In this pathway, the initial dioxygenase attack occurs at the 4,5-carbon position, as previously reported in *Cycloclasticus* and *Mycobacterium* spp. (17, 19). The initial ring-cleavage product of pyrene, 4,5-dicarboxyphenanthrene, could undergo further transformation via reductive decarboxylation and K-region oxidation reactions (31, 35). These reactions are hypothesized to result in the formation of DIPA (P6) (Fig. 4). Moreover, this compound did not create a metabolic bottleneck, as strain ENR6 could utilize DIPA as a sole carbon source and subsequently degraded it via the phthalate pathway. These findings highlighted a previously unrecognized metabolic feature of pyrene catabolism. Given that strain ENR6 was isolated from a petroleum-contaminated site, the DIPA-centered pathway may represent an adaptive response to long-term exposure to HMW-PAHs. Such environments often impose strong selective pressure that promotes metabolic innovation and favors alternative metabolic pathways (36–38).

Although three putative genes (*chr_3347*, *chr_3385,* and *chr_3400*) in the genome of strain ENR6 were predicted to encode RHO α-subunits, only gene *chr_3400*, located in cluster I, was significantly upregulated in response to pyrene degradation. The adjacent genes (*chr_3400-chr_3403*) appear to form a gene cluster encoding a previously uncharacterized RHO system. Nonetheless, heterologous expression and biochemical assays are still required to verify the function of this gene cluster. Moreover, genes *chr_3400-chr_3404* were implicated in the conversion of DIPA in strain ENR6, with *chr_3404* predicted to encode a hydrolase. *Arthrobacter* sp. YC-RL1 was reported to degrade biphenyl with initial dioxygenation on the 2,3-carbon position, followed by ring cleavage and hydrolysis to produce benzoic acid and 2-hydroxypenta-2,4-dienoate (10). The detection of rearrangement and ring fission products, 3,4-benzocoumarin (M1) and phthalic acid (P8), in the degradation culture supported the hypothesis that a similar reaction mechanism may also be involved in the degradation of DIPA by strain ENR6 (Fig. 4).

Earlier studies identified 7,8-benzocoumarin as a lactonization product of (E)-4-(1-hydroxynaphthalen-2-yl)-2-oxobut-3-enoic acid (19, 29). In the present study, the detection of a minor amount of 7,8-benzocoumarin (P5) indicated the occurrence of *meta*-cleavage of 3,4-dihydroxyphenanthrene, likely leading to the formation of 1H2N, even though the latter was not directly detected (Fig. 3). The degradation of 1H2N is mediated either by 1-hydroxy-2-naphthoate dioxygenase producing 2-carboxybenzalpyruvate, as proposed in *Arthrobacter* spp. (11, 38), or by salicylate hydroxylase or functionally equivalent enzymes generating 1,2-naphthalenediol, as reported in some *Rhodococcus* and *Mycobacterium* strains (20, 22).

The presence of 1,2-naphthalenediol (M2), salicylic acid (P7), and phthalic acid (P8) in the 1H2N degradation culture suggested that a hydroxylation reaction occurred, channeling the metabolites into two common “lower pathways” of PAH degradation: the phthalate and salicylate pathways (7, 18, 29). This result was consistent with the RT-qPCR-based expression analysis, which showed that only *chr_3327* and *chr_3341* were upregulated during growth on pyrene within cluster IV. These two genes are predicted to encode a monooxygenase and a dehydrogenase, respectively. RT-qPCR analysis revealed significantly higher transcript levels of cluster IV genes in the culture grown on 1H2N compared to those grown on pyrene (Fig. 7). This suggested that while these genes are involved in the metabolism of 1H2N, their weak induction by pyrene implied this pathway might play a minor role in pyrene degradation in strain ENR6.

### Conclusion

In this study, we identified *Glutamicibacter soli* ENR6 as the first member of its genus capable of degrading PAHs, particularly pyrene. Unlike previously reported pyrene degradation pathways dominated by 1H2N, strain ENR6 primarily degraded pyrene to the phthalate pathway via DIPA as the central intermediate. This finding redefines the role of DIPA in PAH metabolism and highlights a distinct pyrene degradation pathway. Integrated genomic, transcriptomic, and RT-qPCR analyses revealed multiple gene clusters involved in the transformation of pyrene and its intermediates, thereby elucidating the genetic basis underlying the PAH-degrading capacity of strain ENR6. Collectively, our findings expand the current understanding of microbial metabolic diversity in PAH degradation and provide a mechanistic foundation for the potential application of strain ENR6 in PAH bioremediation.

## Data Availability

*Glutamicibacter soli* ENR6 has been deposited in the China Center for Type Culture Collection (CCTCC) under accession number CCTCC M 2024982. The 16S rRNA gene sequence and whole-genome sequence of strain ENR6 have been submitted to NCBI under accession numbers PQ855838 and JBQRUB000000000, respectively. Transcriptomic data (i.e., TPM values for each gene) generated in this study are provided in Table S5.

## SUPPLEMENTAL MATERIAL

Supplemental material aem.02064-25-s0001.docx Tables S1 to S4; Fig. S1 to S6.

Table S5 aem.02064-25-s0002.xls Gene expression levels normalized to transcripts per million (TPM).

ASM does not own the copyrights to Supplemental Material that may be linked to, or accessed through, an article. The authors have granted ASM a non-exclusive, world-wide license to publish the Supplemental Material files. Please contact the corresponding author directly for reuse.
