# Supplementary material for: A distinct 2,2′-diphenic acid-mediated pyrene degradation pathway in a novel PAH degrader, Glutamicibacter soli ENR6
Source: Appl Environ Microbiol. 2026 Mar 3;92(4):e02064-25. doi: 10.1128/aem.02064-25 (PMC13101511; doi:10.1128/aem.02064-25)
Supplement: Supplemental material — Tables S1 to S4; Fig. S1 to S6. [file aem.02064-25-s0001.docx]

**Supplementary Information**

**A distinct 2,2′-diphenic acid-mediated pyrene degradation pathway in a novel PAHs-degrader, *Glutamicibacter soli* ENR6**

Pengrui Zhang ^1^, Haiyang Zhang ^1^, Xinyu Yang ^1^, Yuanli Wu ^1^, Shanshan Dai ^1^, Yanqin Ding ^1 *^, Shanshan Sun^1 *^

^1^ College of Life Sciences and Shandong Engineering Research Center of Plant-Microbial Restoration for Saline-alkali Land, Shandong Agricultural University, Tai’an 271018, China

^#^ Corresponding author:

Yanqin Ding

E-mail: dyq@sdau.edu.cn

Tel. & Fax: +86 0538 8242657

Shanshan Sun

E-mail: shanssun@sdau.edu.cn

Tel. & Fax: +86 0538 8242657

**A Supplemental Tables**

**Table S1** Oligonucleotides used in this study.

**Table S2** Genome properties of *Glutamicibacter soli*.

**Table S3** Average nucleotide identity values (%) calculated between genomes of *Glutamicibacter soli* ENR6 and its closely related species.

**Table S4** Putative genes responsible for the degradation of aromatic compounds in *Glutamicibacter soli* ENR6.

**Table S5** Gene expression levels normalized to transcripts per million (TPM).

**B Supplementary Figures**

**Fig. S1** Utilization of pyrene degradation intermediates by *Glutamicibacter soli* ENR6.

**Fig. S2** Mass spectra of P6-trimethylsilyl (TMS) derivative compared with authentic standard of 2,2'-diphenic acid.

**Fig. S3** GC-MS chromatograms of (A) neutral and (B) acidic metabolites treated with BSTFA-TMCS after 4 d of 2,2’-diphenic acid (50 mg L^-1^) degradation by *Glutamicibacter soli* ENR6.

**Fig. S4** GC-MS chromatograms of (A) neutral and (B) acidic metabolites treated with BSTFA-TMCS after 4 d of 1-hydroxy-2-naphthoic acid (50 mg L^-1^) degradation by *Glutamicibacter soli* ENR6.

**Fig. S5** (A) Differential gene expression in *Glutamicibacter soli* ENR6 cells grown on pyrene versus glucose as the sole carbon source. (B) eggNOG-based functional categories and the number of significantly differentially expressed genes in strain ENR6 cells grown on pyrene and glucose.

**Fig. S6** (A) Neighbor-joining phylogenetic tree based on the amino acid sequence of Chr_3400 from *Glutamicibacter soli* ENR6, constructed using 38 functionally characterized RHO α-subunit sequences. (B) Sequence alignment of Chr_3400 and BphA1f performed using DNAMAN.

**Table S1** Oligonucleotides used in this study.

| **Name** | **Sequences (5’-3’)** | **Description** |
| --- | --- | --- |
| Q3347-F | ACCTGACCCATGAGGAAT | RT-qPCR for gene *chr_3347* |
| Q3347-R | GCCTCGAAGTGGATAATCTG |  |
| Q3341-F | ACTTGGCCATGAGCTTTC | RT-qPCR for gene *chr_3341* |
| Q3341-R | CGGTCACCGACTTTGATATT |  |
| Q3336-F | CTCAACCGTTACGAGACTTC | RT-qPCR for gene *chr_3336* |
| Q3336-R | CCTTCATAGCCATCCAAGAG |  |
| Q3335-F | TCCATGAGTCCCTGTATGAG | RT-qPCR for gene *chr_3335* |
| Q3335-R | TTCGTCGTTGACCGTTTG |  |
| Q3327-F | CAAGCCAGGCAAAGAAGA | RT-qPCR for gene *chr_3327* |
| Q3327-R | GCACATCGACGTTCTCAA |  |
| Q2077-F | GATGAGAAGACCGTGCTG | RT-qPCR for gene *chr_2077* |
| Q2077-R | GCGCAGTAGAAGTTCTCG |  |
| Q285-F | TGAACTACTGGTGGGAGAC | RT-qPCR for gene *chr_285* |
| Q285-R | AGCCCATGTGGTTGAATG |  |
| Q286-F | TACAAGGAACAGCGTGAC | RT-qPCR for gene *chr_286* |
| Q286-R | AAGGTTCGCTGTACATCG |  |
| Q985-F | CTCGACCAATCAGCAGTT | RT-qPCR for gene *chr_985* |
| Q985-R | TGATGGCATGTCCTTGAC |  |
| Q512-F | CACTTCCTGATGAACACTCC | RT-qPCR for gene *chr_512* |
| Q512-R | CATGCCGTCGAAGAAGTT |  |
| Q3411-F | ACCCTGGACCTGTTTATCT | RT-qPCR for gene *chr_3411* |
| Q3411-R | CCAGTTCGGCAAGTGTAAT |  |
| Q3407-F | GTGGACTTGGATACCTGAAAG | RT-qPCR for gene *chr_3407* |
| Q3407-R | GGATGCGGTGATGTTGAA |  |
| Q3404-F | CGACATCATCGGCAACTC | RT-qPCR for gene *chr_3404* |
| Q3404-R | CTCACGGTTCAGGAATTCAA |  |
| Q3403-F | TGCTCTCCATCCTGAAGT | RT-qPCR for gene *chr_3403* |
| Q3403-R | GCTCGTCCTTGAAGATCAC |  |
| Q3402-F | CGGAAAGTTCGGGATGAG | RT-qPCR for gene *chr_3402* |
| Q3402-R | GGGTGGAGTTCAAGAATTGG |  |

**Table S1** Oligonucleotides used in this study (continued).

| **Name** | **Sequences (5’-3’)** | **Description** |
| --- | --- | --- |
| Q3401-F | GTCCAGTGGAACGACGAG | RT-qPCR for gene *chr_3401* |
| Q3401-R | ATCTGCGAGCTTGAGGTG |  |
| Q3400-F | GCTGGACTATGTGATGGAAG | RT-qPCR for gene *chr_3400* |
| Q3400-R | CGACGAAGAACTGGGAATAC |  |

**Table S2** Genome properties of *Glutamicibacter soli*.

| **Content** | **Chromosome** | **Plasmid** |
| --- | --- | --- |
| Size (bp) | 3,856,736 | 72,306 |
| G+C content (%) | 64.24 | 58.18 |
| ORF number | 3578 | 79 |
| ORF total length (bp) | 3,285,516 | 59,520 |
| ORF average length (bp) | 918.25 | 753.42 |
| Coding percentage (%) | 85.19 | 82.32 |
| GC content in ORF region (%) | 65.53 | 59.21 |
| tRNA | 66 | 0 |
| rRNA operon (23S, 16S and 5S) | 19 | 0 |
| ncRNA | 29 | 0 |
|  |  |  |

**Table S3** Average nucleotide identity values (%) calculated between genomes of *Glutamicibacter soli* ENR6 and its closely related species.

|  | ENR6 | *G. soli* | *G. protophormiae* | *G. nicotianae* | *G. mysorens* | *G. halophytocola* | *G. arilaitensis* | *Arthrobacter* sp. Soil736 | *Arthrobacter* sp. Rue61a |
| --- | --- | --- | --- | --- | --- | --- | --- | --- | --- |
| ENR6 | * | **98.52** | 90.14 | 78.26 | 78.51 | 76.62 | 77.02 | 83.25 | 83.87 |
| *G. soli* | **98.49** | * | 90.05 | 77.91 | 78.3 | 76.45 | 76.82 | 83.19 | 83.54 |
| *G. protophormiae* | 90.2 | 90.18 | * | 78.4 | 78.6 | 76.66 | 77.31 | 83.34 | 83.41 |
| *G. nicotianae* | 78.13 | 78.03 | 78.44 | * | 96.98 | 79.27 | 83.55 | 83.48 | 83.54 |
| *G. mysorens* | 78.63 | 78.56 | 78.68 | 97.14 | * | 79.11 | 83.53 | 83.86 | 83.79 |
| *G. halophytocola* | 76.63 | 76.61 | 76.56 | 79.34 | 79.1 | * | 78.93 | 83.2 | 83.76 |
| *G. arilaitensis* | 77.05 | 77.04 | 77.37 | 83.4 | 83.49 | 78.87 | * | 84.86 | 84.87 |
| *Arthrobacter* sp. Soil736 | 83.27 | 83.2 | 83.48 | 83.34 | 83.48 | 85 | 83.21 | * | 82.3 |
| *Arthrobacter* sp. Rue61a | 83.71 | 83.55 | 83.55 | 83.55 | 83.37 | 83.9 | 83.62 | 83.76 | * |

**Table S4** Putative genes responsible for the degradation of aromatic compounds in *Glutamicibacter soli* ENR6.

| **Locus-tag** | **aa** | **Putative protein function** | **Top hit proteins by BLASTP** | | | |
| --- | --- | --- | --- | --- | --- | --- |
|  |  |  | **Organisms** | **Identity (%)/aa** | | **Sequence ID** |
| *chr_126* | 578 | phthalate 4,5-dioxygenase oxygenase reductase | *Pseudomonas putida* | | 27.1/324 | BAA02510.1 |
| *chr_134* | 507 | aldehyde dehydrogenase | *Arthrobacter* sp. YC-RL1 | | 99.6/507 | WP_047117682.1 |
| *chr_135* | 341 | alcohol dehydrogenase | *Micrococcaceae* | | 100/341 | WP_047117683.1 |
| *chr_264* | 287 | quinone oxidoreductase | *Arthrobacter* sp. LS16 | | 96.9/287 | ALD62831.1 |
| *chr_281* | 269 | reductase | *Arthrobacter* sp. AG1021 | | 99.6/269 | WP_120991542.1 |
| *chr_282* | 450 | monooxygenase | *Arthrobacter* sp. AG1021 | | 99.6/450 | WP_120991541.1 |
| *chr_285* | 220 | NADPH-dependent F420 reductase | *Micrococcaceae* | | 83.7/223 | WP_105662393 |
| *chr_286* | 353 | flavin-dependent oxidoreductase | *Micrococcaceae* | | 99.7/353 | WP_181728135 |
| *chr_386* | 199 | alpha/beta hydrolase(*pcad*) | *Glutamicibacter soli* | | 99.5/265 | WP_161448415.1 |
| *chr_387* | 467 | 3-carboxy-*cis*,*cis*-muconate cycloisomerase(*pcab*) | *Arthrobacter* sp. AG1021 | | 99.6/467 | RKS17246.1 |
| *chr_388* | 192 | protocatechuate 3,4-dioxygenase subunit alpha(*pcaG*) | *Micrococcaceae* | | 99.5/192 | WP_047118014.1 |
| *chr_389* | 277 | protocatechuate 3,4-dioxygenase subunit beta(*pcaH*) | *Glutamicibacter soli* | | 100/277 | WP_161448409.1 |
| *chr_390* | 398 | 4-hydroxybenzoate 3-monooxygenase | *Glutamicibacter soli* | | 99.8/398 | WP_161448408.1 |
| *chr_393* | 397 | benzoate transport protein (*benk*) | *Acinetobacter baylyi strain* ATCC 33305 | | 29.8/466 | O30513 |
| *chr_395* | 223 | 3-oxoadipate coa-transferase(*pcai*) | *Arthrobacter* sp. *LS16* | | 98.2/223 | ALD62930.1 |
| *chr_396* | 203 | 3-oxoadipate coa-transferase (*pcaj*) | *Arthrobacter* sp. *LS16* | | 98/216 | ALD62931.1 |
| *chr_397* | 260 | Iclr family transcriptional regulator (*pcar*) | *Glutamicibacter soli* | | 99.2/260 | WP_161448399.1 |

**Table S4** Putative genes responsible for the degradation of aromatic compounds in *Glutamicibacter soli* ENR6 (continued).

| **Locus-tag** | **aa** | **Putative protein function** | **Top hit proteins by BLASTP** | | | | |
| --- | --- | --- | --- | --- | --- | --- | --- |
|  |  |  | **Organisms** | **Identity (%)/aa** | | | **Sequence ID** |
| *chr_512* | 676 | oxidoreductase | *Paeniglutamicibacter gangotriensis* | | 51.9/677 | | WP_149620940 |
| *chr_594* | 488 | aldehyde dehydrogenase | *Bacillus subtilis* | | 31.5/495 | | AAB84440.1 |
| *chr_741* | 496 | aldehyde dehydrogenase | *Mycobacterium* sp. PYR15 | | 34.3/473 | | WP_071948717 |
| *chr_985* | 184 | NAD(P)H-dependent oxidoreductase | *Streptomyces* sp. NPDC053367 | | 73.1/187 | | WP_399653262 |
| *chr_1052* | 425 | salicyloyl-coa 5-hydroxylase | *Streptomyces* sp. | | 43.1/429 | | Q7X281 |
| *chr_1279* | 150 | anthranilate 1,2-dioxygenase | *Burkholderia cepacia* | | 34.9/146 | | Q84BZ0 |
| *chr_1314* | 365 | 3,4-dihydroxyphenylacetate 2,3-dioxygenase | *Bacillus* sp. strain JF8 | | 43.1/299 | | Q8GR45 |
| *chr_1315* | 501 | 5-carboxymethyl-2-hydroxymuconic-semialdehyde dehydrogenase | *Photorhabdus luminescens* | | 46.9/488 | | AA017179.1 |
| *chr_1512* | 289 | catechol 2,3-dioxygenase(*cate*) | *Streptococcus dysgalactiae* | | | 30/234 | WEQ89568.1 |
| *chr_1567* | 336 | NADP-dependent oxidoreductase | *Micrococcaceae* | | 100/336 | | WP_047118998.1 |
| *chr_1610* | 249 | SDR family NAD(P)-dependent oxidoreductase | *Mycolicibacterium pallens* | | 28.7/247 | | WP_071948705.1 |
| *chr_1820* | 286 | SDR family NAD(P)-dependent oxidoreductase | *Mycolicibacterium pallens* | | 28.0/247 | | WP_071948705.1 |
| *chr_1930* | 255 | SDR family NAD(P)-dependent oxidoreductase | *Mycolicibacterium pallens* | | 29.8/247 | | WP_071948705.1 |
| *chr_2077* | 157 | Rieske (2Fe-2S) protein | *Glutamicibacter soli* | | 100/157 | | ALD64280.1 |
| *chr_2274* | 247 | SDR family NAD(P)-dependent oxidoreductase | *Mycolicibacterium pallens* | | 39.5/247 | | WP_071948705.1 |
| *chr_2484* | 261 | Enoyl-coa hydratase | *Glutamicibacter soli* | | 100/261 | | ALQ30059.1 |
| *chr_2485* | 386 | ring-1,2-phenylacetyl-coa epoxidase subunit(*paae*) | *Arthrobacter* sp. AG1021 | | 100/386 | | KLI88611.1 |
| *chr_2486* | 165 | ring-1,2-phenylacetyl-coa epoxidase subunit (*paad*) | *Arthrobacter* sp. AG1021 | | 99.4/165 | | KLI88612.1 |

**Table S4** Putative genes responsible for the degradation of aromatic compounds in *Glutamicibacter soli* ENR6 (continued).

| **Locus-tag** | **aa** | **Putative protein function** | **Top hit proteins by BLASTP** | | | |
| --- | --- | --- | --- | --- | --- | --- |
|  |  |  | **Organisms** | **Identity (%)/aa** | | **Sequence ID** |
| *chr_2487* | 286 | ring-1,2-phenylacetyl-coa epoxidase subunit (*paac*) | *Arthrobacter* sp. AG1021 | | 99.7/286 | KLI88613.1 |
| *chr_2488* | 96 | ring-1,2-phenylacetyl-coa epoxidase subunit (*paab*) | *Arthrobacter* sp. AG1021 | | 100/96 | KLI88614.1 |
| *chr_2489* | 328 | ring-1,2-phenylacetyl-coa epoxidase subunit (*paaa*) | *Arthrobacter* sp. AG1021 | | 100/328 | KLI88615.1 |
| *chr_2665* | 470 | aldehyde dehydrogenase | *Mycobacterium* sp. PYR15 | | 34.1/473 | WP_071948717 |
| *chr_2843* | 89 | 2Fe-2S ferredoxin | *Arthrobacter* sp. MYb213 | | 88.8/113 | PRB68086.1 |
| *chr_2873* | 322 | protocatechuate 3,4-dioxygenase subunit beta | *Arthrobacter* sp. AG1021 | | 98.8/334 | WP_120990960.1 |
| *chr_2876* | 616 | 4-hydroxyphenylpyruvate dioxygenase | *Arthrobacter* sp. AG1021 | | 99/616 | WP_120990964.1 |
| *chr_2877* | 282 | dehydrogenase | *Arthrobacter* sp. YC-RL1 | | 99.3/290 | WP_047120103.1 |
| *chr_2879* | 243 | 3-hydroxybutyrate dehydrogenase | *Arthrobacter* sp. YC-RL1 | | 100/252 | WP_047120105.1 |
| *chr_2880* | 309 | 3-hydroxyisobutyrate dehydrogenase | *Arthrobacter* sp. AG1021 | | 100/309 | WP_053798794.1 |
| *chr 3280* | 264 | 2-keto-4-pentenoate hydratase(*mhpd*) | *Klebsiella pneumoniae* | | 56.1/253 | A6TAC6 |
| *chr 3281* | 324 | acetaldehyde dehydrogenase(*mhpf*) | *Cupriavidus pinatubonensis* | | 66.9/305 | RKS16238.1 |
| *chr 3282* | 335 | 4-hydroxy 2-oxovalerate aldolase(*mhpe*) | *Dechloromonas aromatica* | | 75.8/331 | WP_047120419.1 |
| *chr_3327* | 98 | putative quinol monooxygenase | *Paenarthrobacter* sp. NPDC057981 | | 84.7/103 | WP_387819295 |
| *chr_3330* | 397 | formaldehyde dehydrogenase | *Brachybacterium horti* | | 90.9/397 | GAB4096560.1 |
| *chr_3335* | 495 | benzaldehyde dehydrogenase | *Zafaria cholistanensis* | | 85.0/495 | WP_149957390 |
| *chr_3336* | 293 | isomerase | *Pseudarthrobacter sp. NPDC058329* | | 68.8/292 | WP_387552850.1 |

**Table S4** Putative genes responsible for the degradation of aromatic compounds in *Glutamicibacter soli* ENR6 (continued).

| **Locus-tag** | **aa** | **Putative protein function** | **Top hit proteins by BLASTP** | | | | |
| --- | --- | --- | --- | --- | --- | --- | --- |
|  |  |  | **Organisms** | **Identity (%)/aa** | | | **Sequence ID** |
| *chr_3337* | 258 | SDR family oxidoreductase | *Zafaria cholistanensis* | | 88.8/258 | | WP_149957388.1 |
| *chr_3341* | 356 | alcohol dehydrogenase | *Mycolicibacterium vanbaalenii* PYR-1 | | 30.9/343 | | WP_011777787 |
| *chr_3346* | 337 | oxidoreductase | *Glutamicibacter nicotianae* | | 96.4/337 | | WP_423299549.1 |
| *chr_3347* | 370 | aromatic ring-hydroxylating dioxygenase subunit alpha | *Glutamicibacter* sp. ZJUTW | | 100/370 | | WP_149597683.1 |
| *chr_3367* | 523 | aldehyde dehydrogenase | *Paeniglutamicibacter quisquiliarum* | | 89.1/523 | | MBV1780052.1 |
| *chr 3400* | 348 | Rieske 2fe-2S domain-containing protein(*bpha1*) | *Burkholderia cepacia* | | 20.9/468 | | SQA51808.1 |
| *chr 3401* | 90 | ferredoxin | *Mycolicibacterium porcinum* | | 45.6/92 | | WP_069426098 |
| *chr 3402* | 151 | hypothetical protein | / | | / | | / |
| *chr 3403* | 351 | ferredoxin reductase | *Streptomyces* sp. NBC_01242 | | 66.1/344 | | WP_266904681 |
| *chr 3404* | 300 | 2-hydroxy-6-oxonona-2,4-dienedioate hydrolase | *Arthrobacter* sp. AG1021 | | 100/300 | | WP_047120474.1 |
| *chr 3405* | 394 | Flavin-dependent monooxygenase | *Arthrobacter saudimassilie* | | | 77.4/394 | CEA08718.1 |
| *chr 3407* | 309 | 2,3-dihydroxybiphenyl 1,2-dioxygenase(*bphc*) | *Arthrobacter* sp. YC-RL1 | | 100/308 | | ALQ29294.1 |
| *chr 3411* | 418 | aldolase | *Arthrobacter* sp. YC-RL1 | | 100/418 | | ALQ29291.1 |
| *chr 3416* | 573 | dehydrogenase | *Arthrobacter* sp. AG1021 | | 100/573 | | WP_047120485.1 |


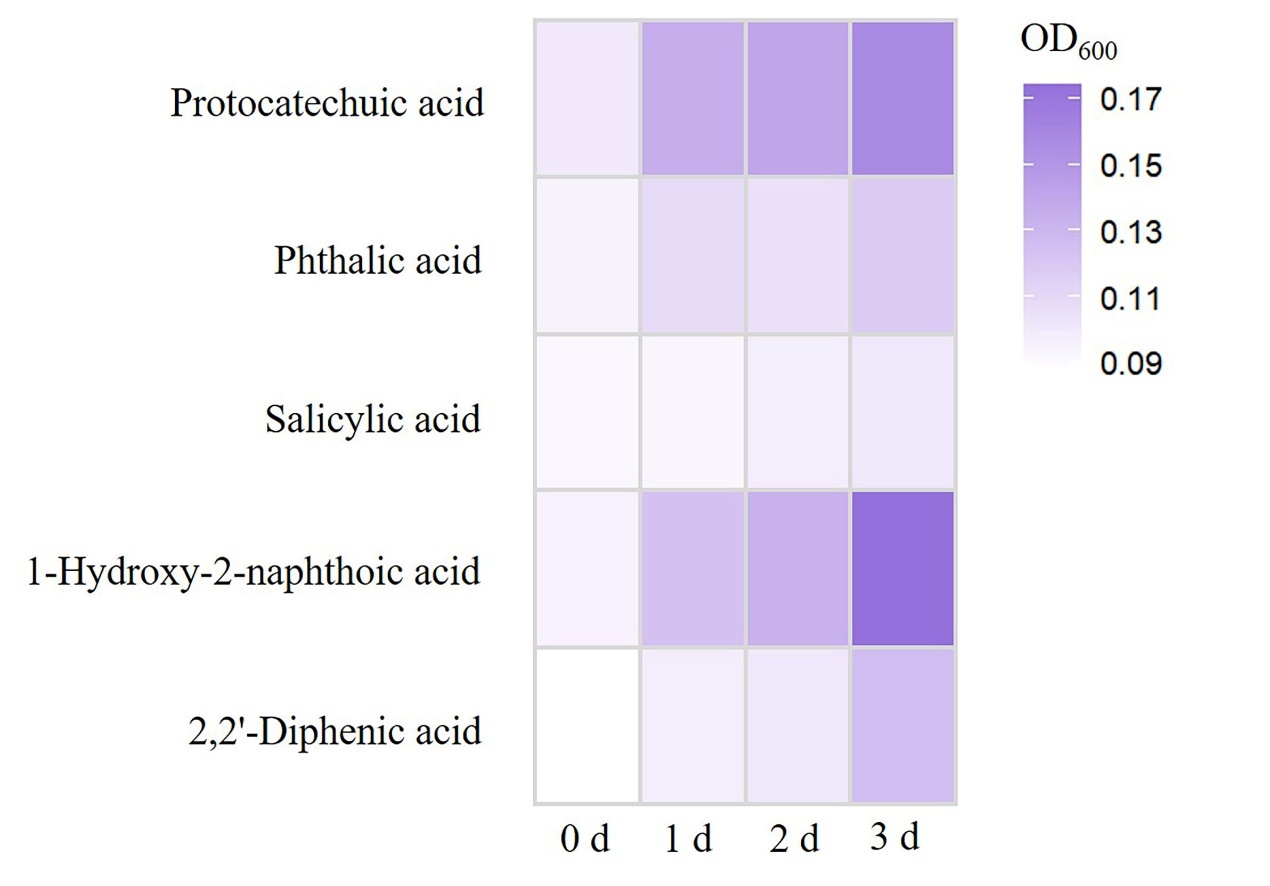
 **Fig. S1** Utilization of pyrene degradation intermediates by *Glutamicibacter soli* ENR6. Strain ENR6 was cultured in MSM supplemented with 50 mg L^-1^ of 2,2′-diphenic acid, 1-hydroxy-2-naphthoic acid, salicylic acid, phthalic acid, or protocatechuic acid as the sole carbon source.


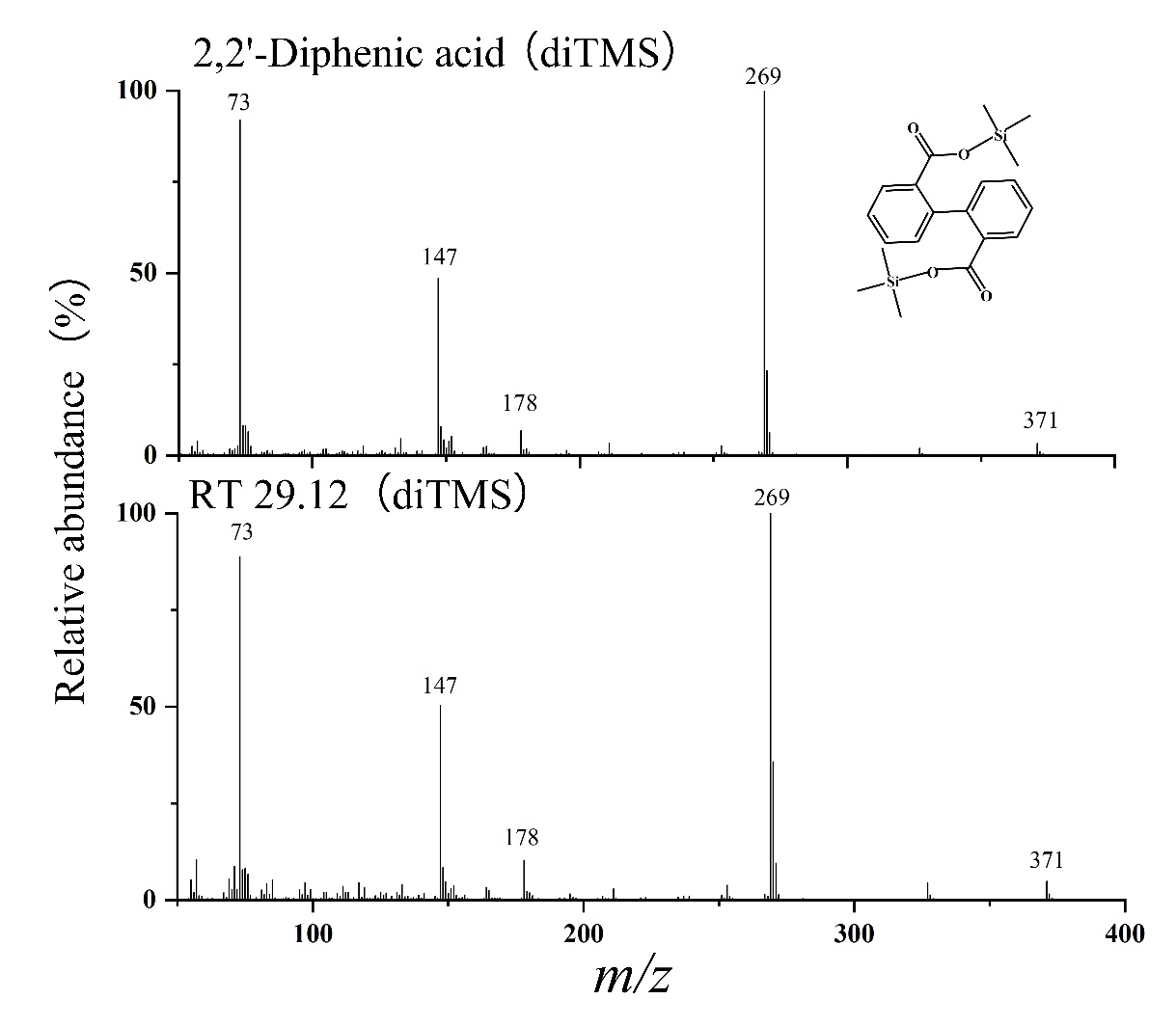


**Fig. S2** Mass spectra of P6-trimethylsilyl (TMS) derivative compared with authentic standard of 2,2'-diphenic acid

**
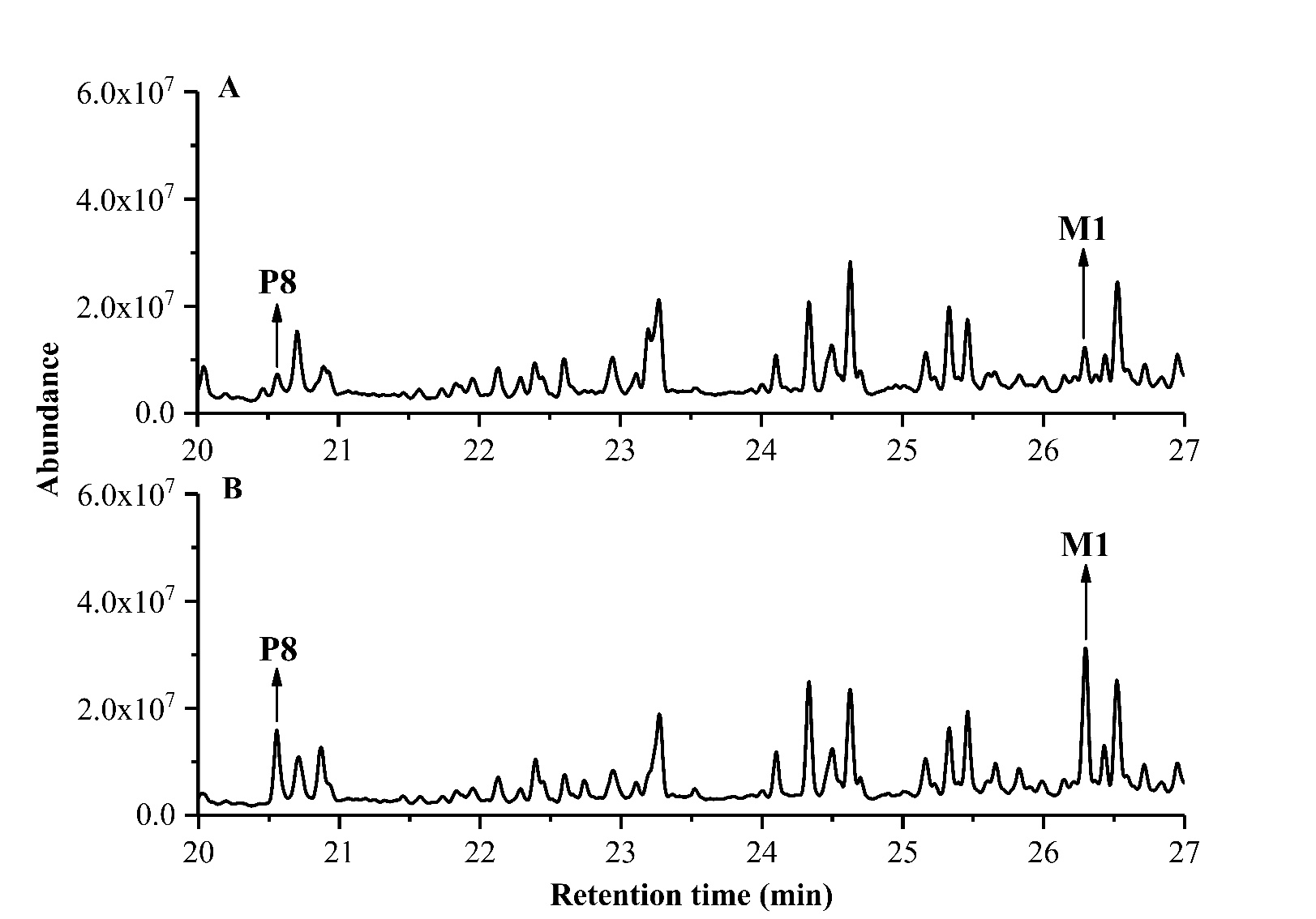
**

**Fig. S3** GC-MS chromatograms of (A) neutral and (B) acidic metabolites treated with BSTFA-TMCS after 4 d of 2,2’-diphenic acid (50 mg L^-1^) degradation by *Glutamicibacter soli* ENR6.

**
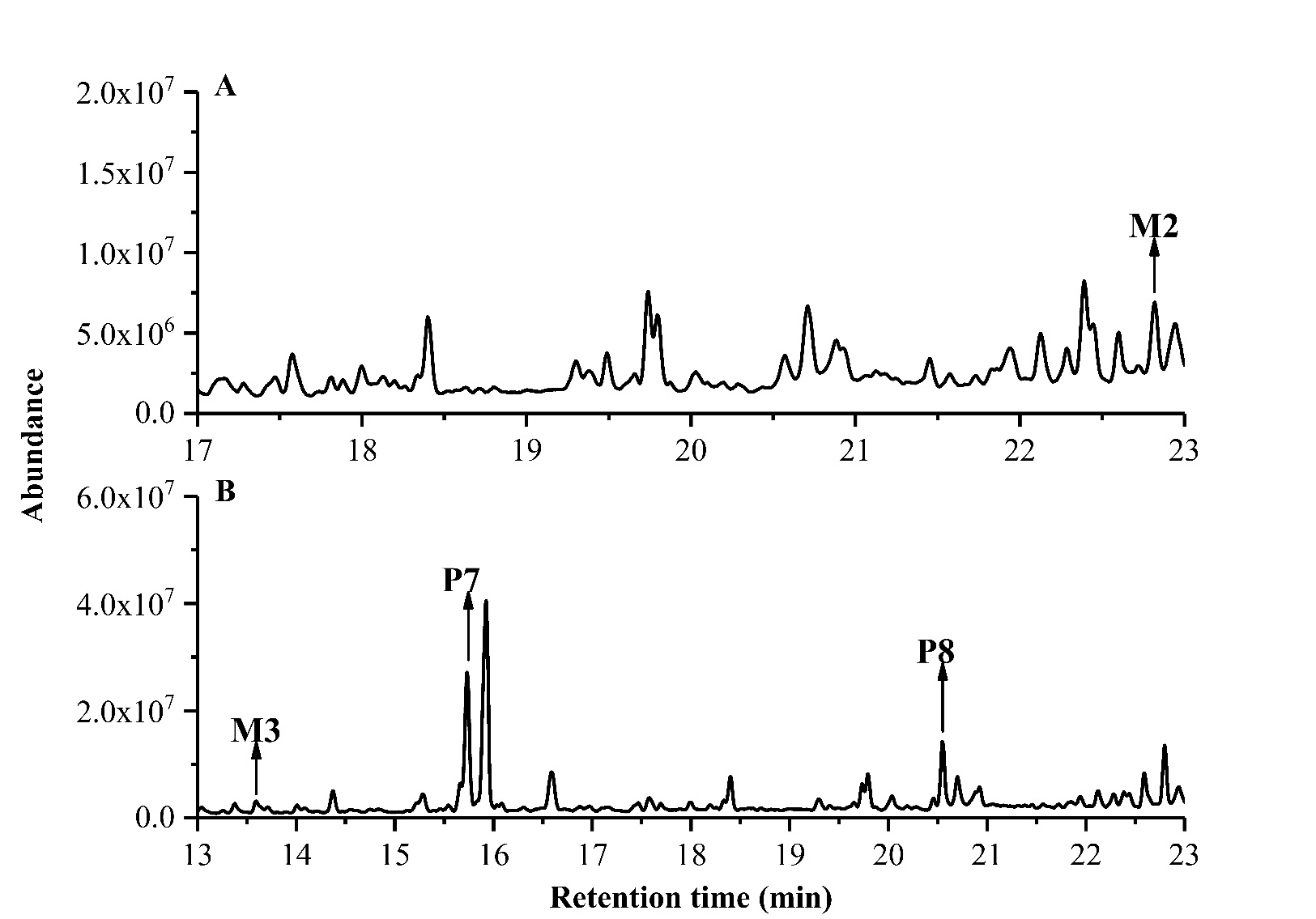
**

**Fig. S4** GC-MS chromatograms of (A) neutral and (B) acidic metabolites treated with BSTFA-TMCS after 4 d of 1-hydroxy-2-naphthoic acid (50 mg L^-1^) degradation by *Glutamicibacter soli* ENR6.


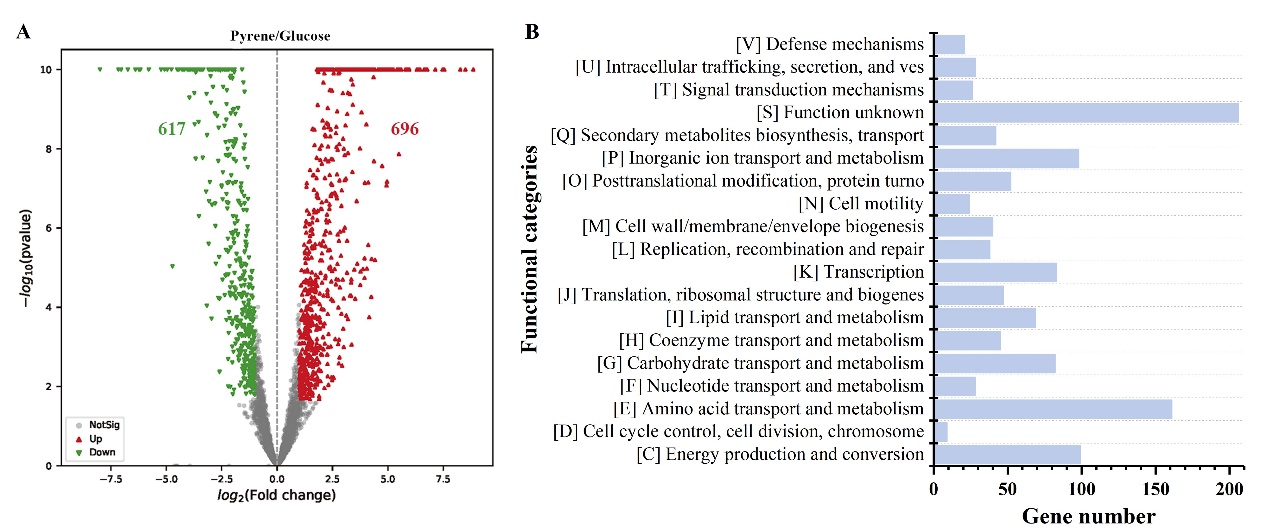


**Fig. S5** (A) Differential gene expression in *Glutamicibacter soli* ENR6 cells grown on pyrene versus glucose as the sole carbon source. (B) eggNOG-based functional categories and the number of significantly differentially expressed genes in strain ENR6 cells grown on pyrene and glucose. The numbers in green and red represent the number of downregulated (log₂FC < -1, FDR < 0.05) and upregulated (log₂FC > 1, FDR < 0.05) genes, respectively.


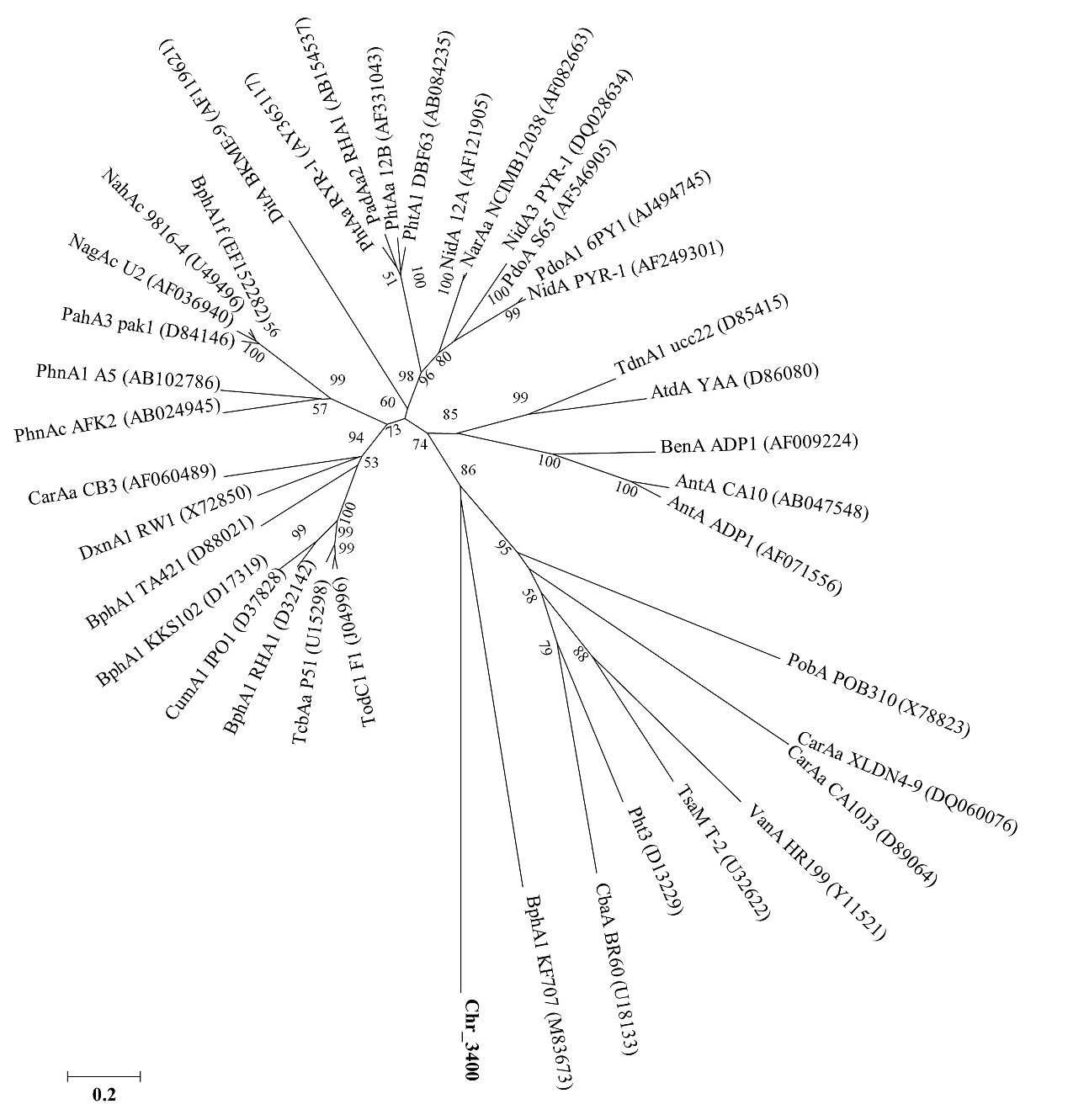


B

A

**
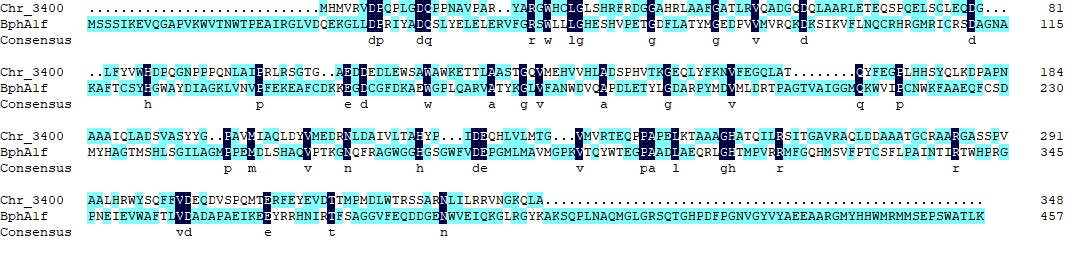
**

**Fig. S6** (A) Neighbor-joining phylogenetic tree based on the amino acid sequence of Chr_3400 from *Glutamicibacter soli* ENR6, constructed using 38 functionally characterized RHO α-subunit sequences. (B) Sequence alignment of Chr_3400 and BphA1f performed using DNAMAN.
